# Kinetically Controlled Self‐Assembly of a Lipophilic Fluorophore with Phenylalanine‐Based Diamides in Artificial Lipid Droplets

**DOI:** 10.1002/smll.202513411

**Published:** 2025-12-29

**Authors:** Miku Naruse, Soichiro Ogi, Keiji Kajiwara, Natsumi Fukaya, Yoshikatsu Sato, Masayasu Taki, Shigehiro Yamaguchi

**Affiliations:** ^1^ Department of Chemistry Graduate School of Science Nagoya University Nagoya Japan; ^2^ Integrated Research Consortium on Chemical Science (IRCCS) Nagoya University Nagoya Japan; ^3^ Institute of Transformative Bio‐Molecules (WPI‐ITbM) Nagoya University Nagoya Japan; ^4^ Institute for Glyco‐core Research (iGCORE) Gifu University Gifu Japan

**Keywords:** fluorescence imaging, lipophilic fluorophore, seed‐initiated assembly, supramolecular polymers, triolein droplets

## Abstract

Lipid droplets (LDs) are vital organelles that govern energy storage and lipid metabolism in cells. The ability to direct the self‐assembly of small molecules into defined nanostructures within LDs would provide opportunities to modulate their functions. Although supramolecular polymerization has been extensively investigated in low‐polarity media, achieving controlled molecular assembly in neutral lipid droplets remains a formidable challenge. Here, we report the design of a lipophilic donor–acceptor‐type fluorophore bearing phenylalanine‐derived diamide units, which undergoes controlled supramolecular polymerization in the neutral lipid triolein. In triolein, the molecule assembles into fibrous aggregates that display intense fluorescence and a pronounced Cotton effect in circular dichroism spectrum. Spectroscopic and microscopic analyses reveal that a seeding strategy accelerates the conversion from a kinetically trapped monomeric state to fibrous assemblies. Furthermore, fluorescence imaging in artificial LDs demonstrates that aggregate size can be tuned through the seeding process.

## Graphical abstract

Fibrous aggregates in triolein are constructed from a lipophilic donor–acceptor fluorophore bearing two phenylalanine‐based diamide units. Spectroscopic analyses and fluorescence imaging revealed a kinetically trapped state persisting for hours, enabling seeded modulation of aggregate size in artificial lipid droplets encapsulating triolein within a surfactant shell.

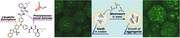

## 1 Introduction

Supramolecular polymers, formed through 1D assembly of small molecules via non‐covalent interactions, exhibit behaviors analogous to those of covalent polymers [1, 2, 3, 4, 5, 6, 7, 8]. A distinguishing feature of these materials is their ability to undergo reversible polymerization and depolymerization, enabling the development of stimuli‐responsive and self‐healing systems [9, 10], In recent years, supramolecular polymers have gained considerable attention as tools for modulating cellular activities, with particular interest in their use within living cells [11, 12, 13, 14, 15, 16, 17, 18, 19, 20, 21, 22, 23, 24]. In particular, the in situ formation of nanofibers within cellular organelles holds great promise for achieving selectively and precisely control over organelle activity. For instance, mitochondrial‐targeting ligands conjugated with self‐assembling peptides have been shown to form nanofibers that accumulate within mitochondria, where they trigger cancer cell death and suppress cancer cell proliferation [25].

**FIGURE 1:**
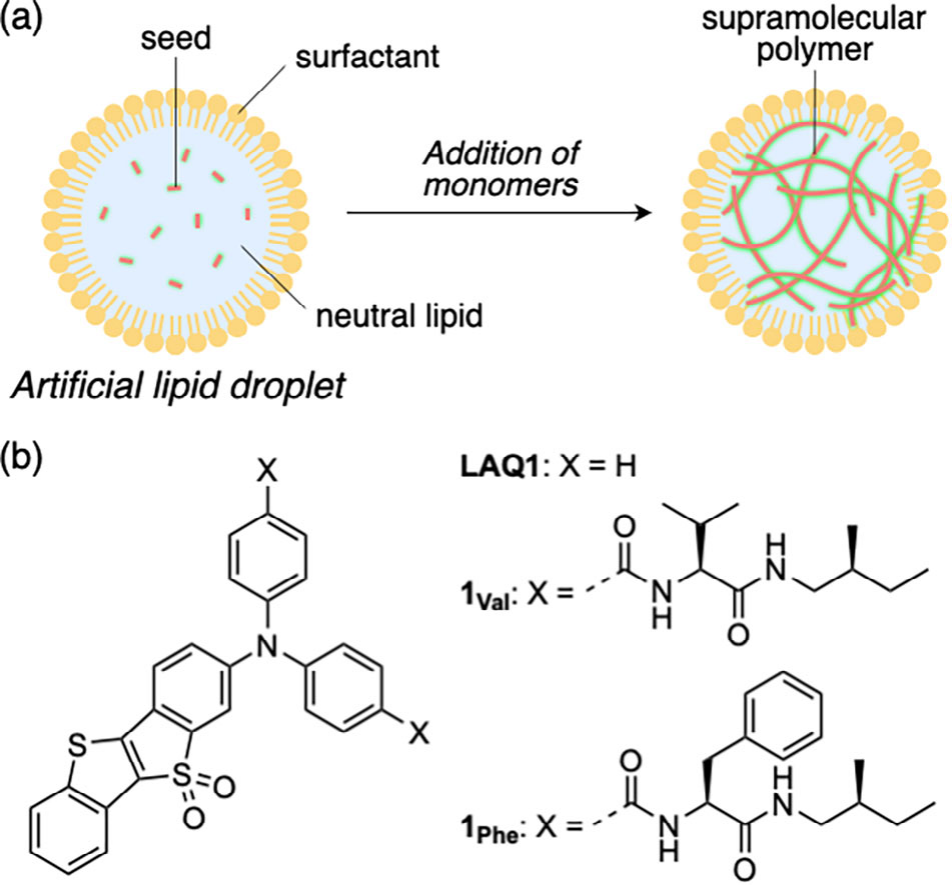
(a) Schematic representation of seeded polymerization in an artificial lipid droplet; (b) chemical structures of LAQ1 and its diamide‐conjugates 1_Val_ and 1_Phe_ used in this study.

Lipid droplets (LDs) are essential cellular organelles that store neutral lipids, such as triacylglycerols (TG) and cholesterol esters (CE), and play a pivotal role in maintaining cellular homeostasis [26, 27]. These lipid reservoirs facilitate lipid exchange with other organelles through membrane contact sites, thereby contributing to various physiological processes [28, 29, 30]. The dynamics of LDs are closely linked to energy metabolism and lipid homeostasis, making them integral to the pathogenesis of metabolic diseases like obesity and diabetes [31, 32, 33, 34, 35, 36]. Despite significant progress in understanding LD biology, controlling their function remains a major challenge, particularly in modulating their dynamics in living cells. Supramolecular polymerization of small relevant molecules in LDs could provide a novel approach to manipulating LD dynamics. However, while substantial advances have been made in designing molecular systems for supramolecular polymerization in low‐polarity solvents, achieving precise molecular assembly within neutral lipid droplets remains a significant hurdle. Developing methods for supramolecular polymerization compatible with such environment as LDs is therefore crucial (Figure 1a).

To achieve this, molecules are required to possess three key features: (1) appropriate lipophilicity to ensure their localization within LDs, (2) strong self‐assembling ability to overcome solvation effects in neutral lipids, and (3) intense fluorescence in the aggregated state to enable imaging. Although not originally designed for this purpose, we previously developed super‐photostable fluorescent probe LAQ1 (Figure 1b), which selectively stains intracellular LDs [37]. LAQ1 exhibits exceptional resistance to photobleaching, enabling the visualization of ultrasmall LDs via super‐resolution simulated emission depletion (STED) microscopy and the long‐term tracking of LD dynamics. In parallel, to achieve controlled self‐assembly, we incorporated amino‐acid‐based diamides to various π‐conjugated frameworks as hydrogen‐bonding motifs, thereby providing a robust scaffold for produced 1D, kinetically controlled supramolecular assemblies [38, 39, 40, 41, 42]. In the present study, we integrate these two design strategies by conjugating LAQ1 with diamide chains onto the diphenylamino moiety (Figure 1b) to construct lipophilic fluorophores capable of controlled self‐assembly within neutral lipids.

In the resulting LAQ–diamide conjugates, preserving the intrinsic lipophilicity of LAQ1 is crucial to ensure its spontaneous localization within LDs. Lipophilicity was estimated using the miLogP values calculated with Molinspiration software [43]. Compared to LAQ1 (miLogP = 7.25), the introduction of the diamide groups tend to decrease miLogP, for example, 4.89 for a LAQ–alanine‐based diamide conjugate (Figure S1). To enhance LD selectivity, we sought to optimize lipophilicity by varying the amino acid residues and side chains, which led to the design of valine‐ or phenylalanine‐based diamide derivatives having chiral alkyl chains, 1_Val_ and 1_Phe_, with miLogP values of 9.30 and 10.2, respectively. The aggregation and luminescence properties of these compounds were then evaluated in triolein, a representative TG. To mimic LD‐like conditions, artificial LDs were formed by encapsulating triolein within a surfactant shell, enabling the assessment of self‐assembly through fluorescence imaging.

## 2 Results and Discussion

### 2.1 Synthesis

**SCHEME 1:**
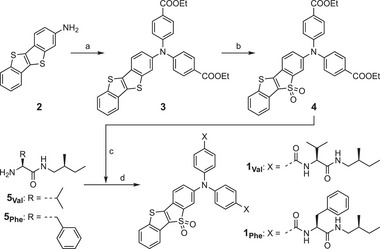
Synthesis of LAQ–diamide conjugates 1_Val_ and 1_Phe_. *Reagents and conditions*: (a) ethyl 4‐iodobenzoate, CuI, K_2_CO_3_, in *o*‐dichlorobenzene, 195°C, 63 h; (b) *m*CPBA, in 1,2‐dichloroethane, rt, 5 h; (c) NaOH, in THF/water, rt, 15 h; (d) *N*‐TBTU, DIEA, in DMF, rt, 23 h.

The LAQ–diamide conjugates were synthesized via the amidation of carboxylated derivatives of LAQ1 with chiral amines (Scheme 1). Thus, starting from 2‐amino‐[1]benzothieno[3,2‐*b*][1]benzothiophene (2) [44], Ullmann coupling reaction with ethyl 4‐iodobenzoate afforded 3 in 92% yield. Oxidation of 3 with *m*CPBA gave 4, which upon treatment with an aqueous NaOH solution followed by amidation with the corresponding chiral amines 5_Val_ and 5_Phe_ produced 1_Val_ and 1_Phe_ in 73% and 61% yields, respectively. A mono(diamide)‐substituted compound 1_Phe_ *'* was synthesized as a reference compound in the same manner as 1_Phe_, except that a mixture of iodobenzene and ethyl 4‐iodobenzoate was used in the Ullmann coupling step. Full details of the synthesis and characterization of these fluorescent dyes are provided in the Supporting Information.

### 2.2 Self‐Assembly Behavior in Triolein

**FIGURE 2:**
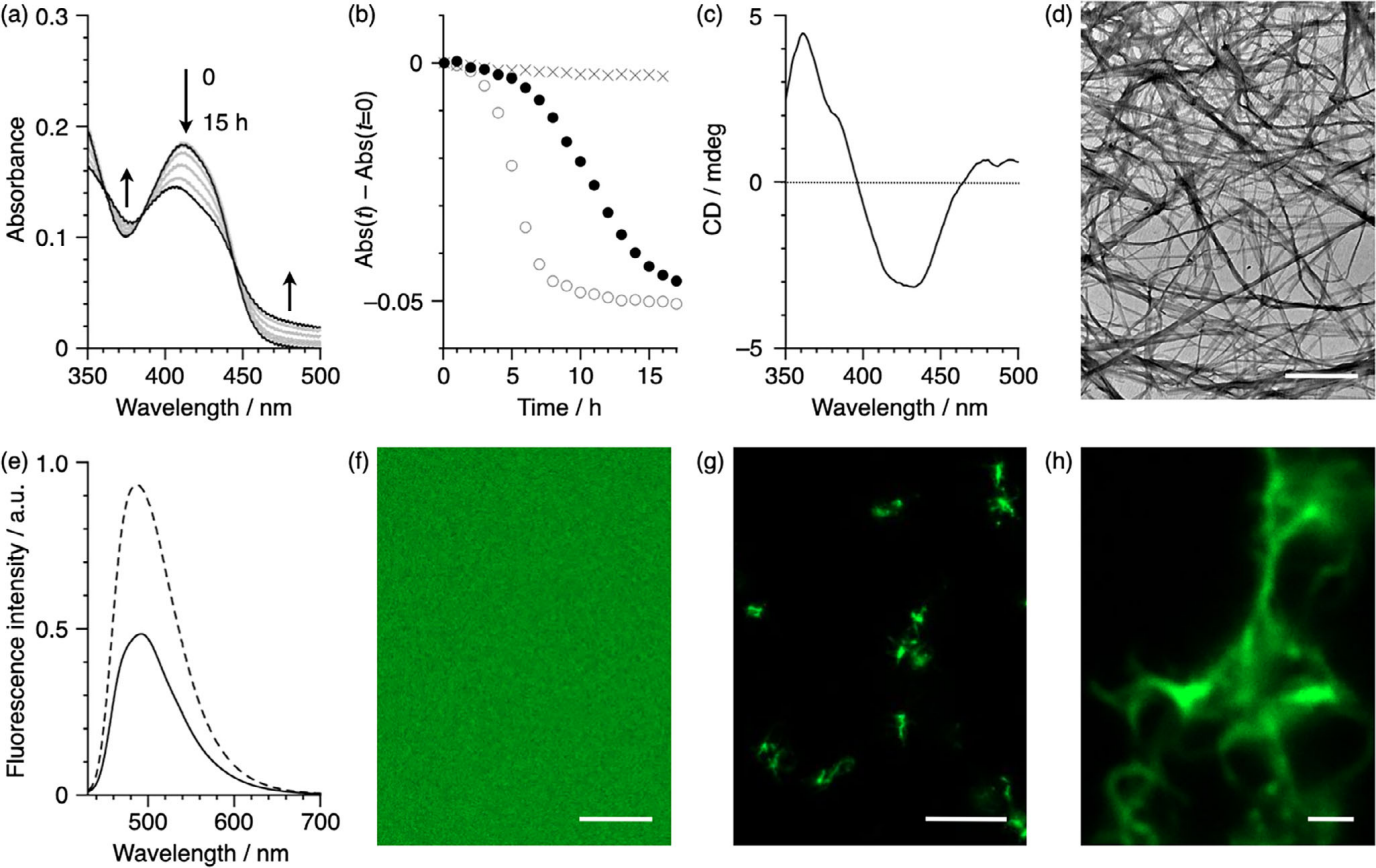
(a) Time‐dependent changes in the absorption spectra of 1_Phe_ in triolein after fast cooling from 370 to 293 K at a total concentration of 1.0 × 10^−5^ m. (b) Time courses of the absorbance changes at 411 nm of 1_Phe_ in triolein at 0.5 × 10^−5^ m (crosses), 1.0 × 10^−5^ m (filled circles), and 1.5 × 10^−5^ m (open circles). (c) A CD spectrum of 1_Phe_ in triolein (1.0 × 10^−5^ m) obtained 15 h after fast cooling from 370 to 293 K. (d) A TEM image (scale bar = 1 µm) of 1_Phe_ aggregates prepared in di‐*n*‐butyl ether. (e) Fluorescence spectra of 1_Phe_ in the monomeric state (dashed line) and in the aggregated state (solid line) in triolein (1.0 × 10^−5^ m), recorded immediately after and 18.5 h after fast cooling from 370 to 293 K, respectively. (f–h) Confocal images of 1_Phe_ in the monomeric state (f, scale bar = 10 µm) and the aggregated state (g, scale bar = 10 µm; h, scale bar = 1 µm) in triolein; conditions: *c*_T_ = 1.0 × 10^−5^ m, *λ*_ex_ = 405 nm, *λ*_em_ = 470–540 nm.

Initially, the self‐assembly behavior of 1_Phe_ in triolein was investigated by monitoring time‐dependent changes in the UV–vis absorption spectrum. For sample preparation, triolein was added to a solution of 1_Phe_ in toluene (1.0 × 10^−5^ m) until the toluene content reached 50 vol%, after which toluene was removed under reduced pressure. Prior to the measurements, the resulting solution was heated and then cooled to 293 K. The triolein solution of 1_Phe_ exhibited time‐dependent spectral changes, including a hypsochromic shift of the absorption band from 411 to 400 nm (Figure 2a). The isosbestic points observed at 357, 394, and 457 nm indicate a single transition to an energetically stable state. Monitoring the absorbance change at 411 nm revealed a sigmoidal transition, characteristic of an autocatalytic process (Figure 2b, filled circles). The transition rate displayed a pronounced dependence on concentration, accelerating at 1.5 × 10^−5^ m, but slowing at 0.5 × 10^−5^ m (Figure 2b; Figure S2). This tendency has been observed in systems that develop metastable states during the process of spontaneous assembly from a molecularly dispersed state into an aggregate state [45]. According to previous studies on the self‐assembly of the diamide‐substituted π‐systems [38, 39, 40, 41, 42], the folded structure formed by an intramolecular hydrogen bond of the diamide moiety is a possible metastable state in this system. However, unlike commonly used low‐polarity solvents, in triolein, self‐assembly through intermolecular hydrogen bonding between diamide units might be influenced by the high viscosity (44.7 mm^2^·s^−1^) and hydrogen‐bonding acceptor carbonyl groups of triolein [46]. It should be noted that the absorption spectral changes were accompanied by the appearance of bisignate CD signals with positive and negative maxima at 361 and 433 nm, respectively (Figure 2c). These spectral features suggest the transition from the monomeric state to an aggregate state with chiral excitonic coupling of the transition dipole moments [47].

The role of the amino‐acid‐based diamide moieties was examined by comparing the spontaneous self‐assembly of 1_Val_ or 1_Phe_ *'* with that of 1_Phe_. The time‐dependent absorption measurements of 1_Val_ in triolein were performed under the same conditions as those for 1_Phe_. However, no detectable spectral changes indicative of self‐assembly were observed even at concentrations as high as 1.1 × 10^−4^ m (Figure S3a,b). This result demonstrates that 1_Val_ has a low propensity for spontaneous nucleation in triolein, despite possessing two diamide units, and the nature of the amino acid residues plays a crucial role in determining the nucleation kinetics. Meanwhile, 1_Phe_ *'*, which contains only one diamide unit, showed no sigmoidal transition in absorbance even after 30 h (Figure S3c,d). These results indicate that the introduction of two phenylalanine‐based diamide units to the LAQ1 scaffold is effective in biasing the equilibrium toward the formation of supramolecular assemblies.

Microscopic studies were conducted to gain insight into the morphology of the 1_Phe_ aggregates. Transmission electron microscopy (TEM) is a powerful tool for observing aggregate structures. However, the high boiling point of triolein makes it difficult to prepare dried samples for observation. Therefore, aggregates of 1_Phe_ were prepared using di‐*n*‐butyl ether (DBE), which has a dielectric constant (*ε* = 3.06 at *T* = 25°C) comparable to that of triolein (*ε* = 3.20 at *T* = 25°C) but a lower boiling point [48]. The resulting solution exhibited a hypsochromic shift of the absorption band and an induced CD spectrum, similar to those observed for the triolein solution (Figure S4a,b). TEM observations confirmed that 1_Phe_ forms fibrous aggregates in DBE (Figure 2d). Furthermore, to verify that the fibrous aggregates observed by TEM are stabilized through intermolecular hydrogen bonding, we measured the FT‐IR spectrum of the aggregated sample prepared in DBE. The spectrum exhibited a broadened N─H stretching band at 3293 cm^−1^ and a C═O stretching band at 1631 cm^−1^ (Figure S4c,d), both of which appear at lower wavenumbers compared with the monomeric state [42]. These shifts are characteristic signatures of diamide‐based intermolecular hydrogen bonding, confirming that the fibrous aggregates of 1_Phe_ in low‐polarity media are formed through hydrogen‐bond‐driven association between the phenylalanine‐derived diamide units. Focusing on the luminescence properties of the aggregate state, the fluorescence spectrum of 1_Phe_ aggregates retained nearly the same shape as in the monomeric state, while the quantum yield decreased from 0.73 to 0.36, resulting in approximately half the emission intensity (Figure 2e). Taking advantage of the still appreciable fluorescence in the aggregate state, we envisioned that fluorescence imaging enables direct visualization of the supramolecular nanostructures formed in triolein. After heating and then cooling to room temperature, a confocal image of the solution of 1_Phe_ in triolein, taken by confocal laser scanning microscopy (CLSM), exhibited uniform fluorescence distributed across the entire field of view (Figure 2f). In contrast, after standing the solution for 18 h, a number of strongly emitting objects were observed in the solution (Figure 2g). Magnification of these objects revealed fibrous nanostructures (Figure 2h), which should correspond to supramolecular polymer formed in triolein.

### 2.3 Cooperative and Seed‐Initiated Assembly in Triolein

**FIGURE 3:**
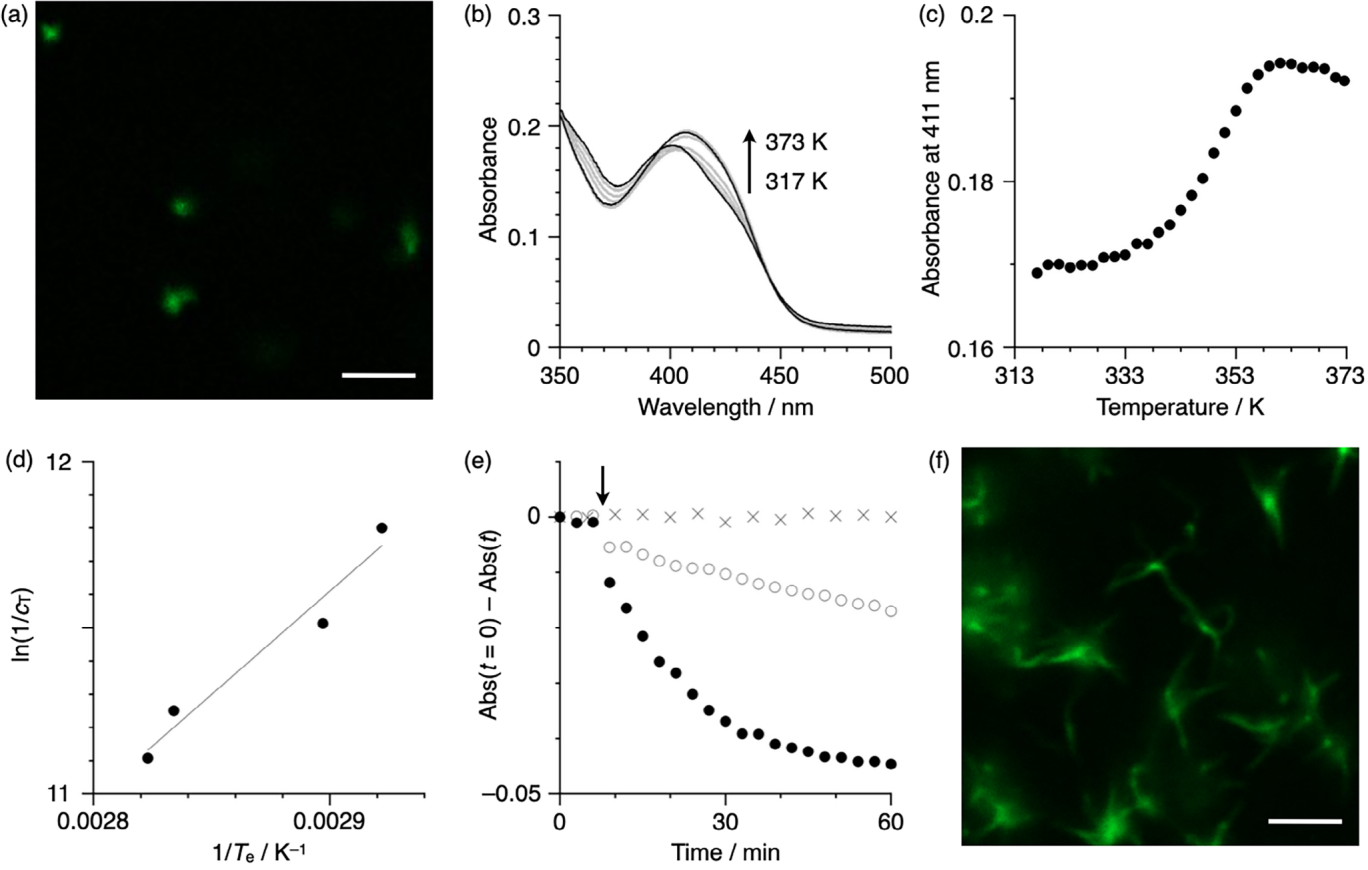
(a) Confocal image of 1_Phe_ seeds (scale bar = 3 µm) in triolein. Conditions: *c*_T_ = 1.0 × 10^−5^ m, *λ*_ex_ = 405 nm, *λ*_em_ = 470–540 nm. (b) UV–vis absorption spectral changes of 1_Phe_ in triolein (1.0 × 10^−5^ m) upon increasing temperature from 317 to 373 K at a rate of 1 K min^−1^. (c) Temperature‐dependent changes in absorbance at 411 nm. (d) Plot of natural logarithm of the reciprocal *c*_T_ as a function of the reciprocal *T*_e_ obtained from heating experiments of 1_Phe_ in triolein at different concentrations. (e) Time‐dependent absorbance at 411 nm before and after addition of 1_Phe_ seeds in triolein (0.6 mL for filled circles; 0.1 mL for open circles; 1.0 × 10^−5^ m) to 1_Phe_ monomers in triolein (2.4 mL for filled circles; 2.0 mL for open circles; 1.0 × 10^−5^ m) at the time indicated by the arrow, or without seeds (crosses). (f) Confocal image of the resulting aggregates obtained after seeded polymerization (scale bar = 3 µm). Conditions: *c*_T_ = 1.0 × 10^−5^ m, *λ*_ex_ = 405 nm, *λ*_em_ = 470–540 nm.

The mechanism of the self‐assembly was investigated in detail by monitoring the temperature‐dependent changes in the UV–vis absorption spectra. For sample preparation, a solution of 1_Phe_ in triolein was heated and subjected to sonication for 1 h in a water bath at 293 K. Compared with the 1_Phe_ aggregates in triolein prepared by aging for 18 h (Figure 2g,h), the aggregates obtained by sonication were shorter and more fragmented (Figure 3a), exhibiting a similar absorption band with a maximum at 400 nm. This suggests that ultrasonic treatment is effective in reducing the aging time to less than an hour. Upon heating the solution from 317 to 373 K at a rate of 1 K min^−1^, the absorbance at 411 nm increased, indicating the disassembly of aggregates into monomers (Figure 3b). The critical temperature (*T*_e_) was determined from this heating curve, where the temperature dependence of the absorbance follows a non‐sigmoidal transition with a *T*_e_ of 357.5 K (Figure 3c). The *T*_e_ values decreased from 367.3 to 354.3 K when the total concentration of 1_Phe_ was reduced from 1.5 × 10^−5^ to 7.5 × 10^−6^ m (Figure S5). A van't Hoff plot based on the natural logarithm of the reciprocal *c*_T_ as a function of the reciprocal *T*_e_ showed a linear relationship (Figure 3d) [49, 50], from which the standard enthalpy (Δ*H*° = –51.5 kJ mol^−1^) and entropy (Δ*S*° = –47.6 J mol^−1^ K^−1^) changes for the elongation were determined. The Δ*G*° value for 293 K, calculated from the obtained thermodynamic parameters, is –37.6 kJ mol^−1^, which is sufficient for more than 95% of 1_Phe_ molecules to be in an aggregated state.

The cooperative formation of 1_Phe_ aggregates was further supported by the experimental observation that supramolecular polymerization was initiated by the addition of fragmented aggregates (seeds). Seeds were prepared by applying ultrasonic treatment to a hot triolein solution of monomeric 1_Phe_ for 1 h in a water bath. During the lag time observed in the early stage of spontaneous assembly, the addition of seeds resulted in a decrease in absorbance at 411 nm without a lag time (Figure 3e, open circles; Figure S6a), producing elongated supramolecular polymers as confirmed by fluorescence imaging (Figure 3f). The polymerization was accelerated as the amount of seeds increased (Figure 3e, filled circles; Figure S6b), whereas no change in absorption was observed in the absence of seeds (Figure 3e, crosses). Accordingly, these findings confirm that the seed‐initiated assembly of 1_Phe_ proceeded successfully in triolein.

### 2.4 Spontaneous and Controlled Assembly in Artificial Lipid Droplets

**FIGURE 4:**
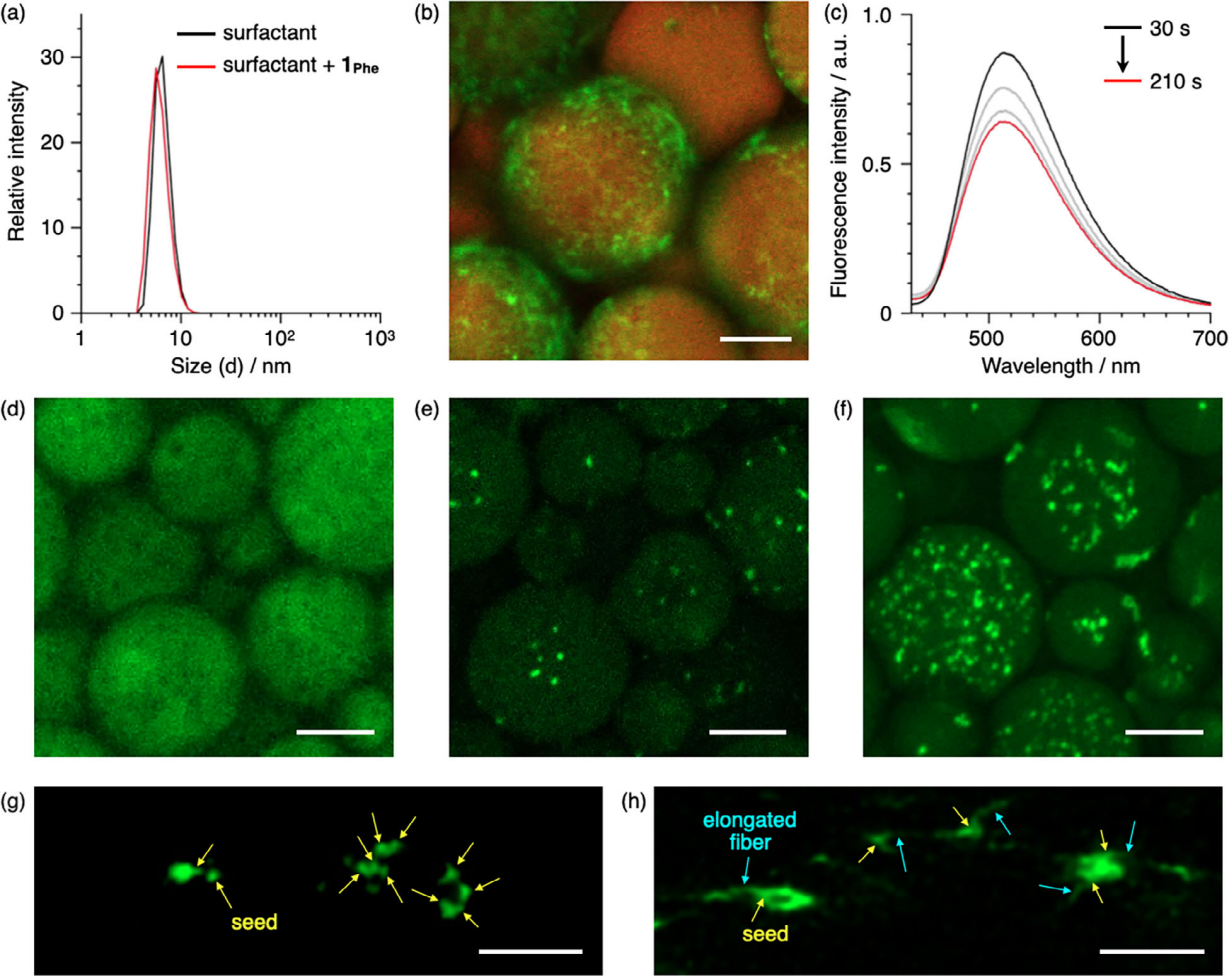
(a) Number size distribution of a surfactant‐only aqueous solution (3 wt.%) and a mixture of 1_Phe_ in DMSO (1.0 × 10^−4^ m; 0.28 mL) and surfactant in water (3 wt.%; 2.8 mL; red line), determined by dynamic light scattering (DLS). (b) Confocal laser scanning microscopy (CLSM) image of 1_Phe_ aggregates (1.0 × 10^−5^ m) with Nile red (6.7 × 10^−6^ m) in artificial LDs; green channel for 1_Phe_: *λ*_ex_ = 405 nm, *λ*_em_ = 470–540 nm; red channel for Nile red: *λ*_ex_ = 556 nm, *λ*_em_ = 650–695 nm; scale bars: 50 µm. (c) Changes in fluorescence intensity of 1_Phe_ in aqueous phase in the presence of artificial LDs before and after 210 s of vortexing. (d,e) CLSM images of 1_Phe_ monomers (d) and seeds (e) after 210 s of vortexing and 2 h of standing. (f) CLSM image of 1_Phe_ aggregates obtained by addition of monomeric 1_Phe_ in DMSO to water containing artificial LDs with pre‐formed 1_Phe_ seeds followed by vortexing for 210 s and allowing to stand for 2 h; *λ*_ex_ = 405 nm, *λ*_em_ = 470–540 nm; scale bars: 30 µm. (g) High‐magnification CLSM image of 1_Phe_ seeds within an artificial LD; *λ*_ex_ = 405 nm, *λ*_em_ = 480–640 nm. and (h) STED image of 1_Phe_ aggregates obtained after adding monomeric 1_Phe_ in DMSO to water containing artificial LDs with pre‐formed 1_Phe_ seeds; *λ*_ex_ = 405 nm, *λ*_STED_ = 592 nm, *λ*_em_ = 480–585 nm; scale bars: 2 µm.

Given our understanding of the self‐assembly behavior of 1_Phe_ in triolein, we next investigated the self‐assembly in an artificial LD environment. As a preliminary demonstration, we examined whether 1_Phe_ could be incorporated into triolein droplets surrounded by a surfactant shell, thereby mimicking the uptake process of cellular LDs. Surfactant‐coated triolein droplets (artificial LDs) were prepared by mixing triolein with an aqueous solution of Brij58 surfactant [51] (3 wt.%) and vortexing for 10 s. The resulting droplets floated in the aqueous phase. Monomeric 1_Phe_ in DMSO was then introduced into the aqueous phase and vortexed for 210 s. In the absence of droplets, the mixture of surfactant and 1_Phe_ exhibited a dispersion pattern identical to that of the surfactant‐only solution (Figure 4a; Figure S7). Under these conditions, the absorption maximum and intensity of 1_Phe_ were essentially identical to those of monomeric 1_Phe_ in triolein, and no CD signal was detected (Figure S8a,b), indicating that 1_Phe_ is molecularly dispersed within Brij58 micelles. Additionally, the fluorescence maximum appeared at 518 nm (Figure S8c, red line), which lies between the emission wavelengths observed in triolein and DMSO, consistent with solubilization of 1_Phe_ in a micellar environment of intermediate polarity. In contrast, when droplets were present, fluorescence imaging revealed a detectable fluorescence within the droplets (Figure S9), confirming successful transfer of 1_Phe_ into the triolein core. These results demonstrate the feasibility of 1_Phe_ uptake into artificial LDs.

To gain deeper insight into the self‐assembly behavior of 1_Phe_ within artificial LDs under defined conditions, droplets were prepared directly from triolein solutions containing a known concentration of monomeric 1_Phe_. At 1.0 × 10^−5^ m, fluorescence imaging performed 10 min after preparation revealed homogeneous emission throughout the droplets, indicating that 1_Phe_ remained monodisperse (Figure S10b, left). Notably, no aggregates were observed even after 4 h, whereas fibrous aggregates became evident after 1 d of standing (Figure S10b, right), revealing the presence of an induction phase before spontaneous self‐assembly commenced. Co‐staining with Nile red, a standard probe for intracellular LDs, further confirmed that 1_Phe_ aggregates localized within the artificial LDs (Figure 4b; Figure S11). The assembly kinetics were strongly concentration‐dependent: no aggregates appeared even after 1 day at 0.5 × 10^−5^ m (Figure S10a), whereas aggregates formed within 4 h at 1.5 × 10^−5^ m (Figure S10c). This concentration‐dependent behavior, including the presence of an induction phase prior to aggregate formation, closely resembled that observed in bulk triolein (Figure 2b). These findings indicate that the kinetics of self‐assembly of 1_Phe_ are intrinsic and remain unaffected by encapsulation within the surfactant‐stabilized droplets. In contrast, 1_Val_ showed no detectable self‐assembly within artificial LDs. Confocal imaging performed immediately after droplet preparation, after 4 h, and after 1 d consistently revealed uniform fluorescence without any localized emissive domains or fibrous structures (Figure S12). This behavior matches our findings in bulk triolein, where 1_Val_ did not undergo spontaneous self‐assembly even at 1.1 × 10^−4^ m (Figure S3a,b), indicating that its much weaker tendency to self‐assemble compared with 1_Phe_ also persists in the LD environment.

Finally, the regulation of self‐assembly in artificial LDs through seed‐initiated growth was investigated. Monomeric 1_Phe_ in DMSO (*c* = 1.0 × 10^−4^ m) was added to water containing artificial LDs with pre‐formed 1_Phe_ seeds (*c* = 1.0 × 10^−5^ m), followed by vortexing and measuring fluorescence spectra. The fluorescence intensity of the aqueous phase at 515 nm decreased by approximately 25% after 210 s of vortexing compared to pre‐treatment levels (Figure 4c), indicating incorporation of monomeric 1_Phe_ into the droplets. Prior to imaging, the sample was allowed to stand at 293 K for 2 h, a duration sufficient to initiate assembly as validated in bulk triolein experiments (Figure 3e). Under these conditions, both monomeric 1_Phe_ and the morphology of seeds in artificial LDs remained unchanged (Figure 4d,e). By contrast, at a seed‐to‐monomer ratio of 1:5 (*v*/*v*), the size of 1_Phe_ aggregates in artificial LDs increased relative to the seeds alone (Figure 4f). To further elucidate the morphology of the assemblies formed within artificial LDs, we performed high‐resolution fluorescence microscopy. CLSM imaging at higher magnification revealed diffraction‐limited, particle‐like emissive domains corresponding to the pre‐formed seeds (Figure 4g). Their rapid Brownian motion within the droplets was captured in a time‐lapse CLSM imaging (Movie S1), providing direct evidence for the nanoscale, highly mobile nature of these seed species. Upon addition of monomeric 1_Phe_, these emissive objects exhibited reduced diffusional mobility, suggesting supramolecular growth. Under these conditions, STED microscopy enabled direct visualization of elongated, fiber‐like nanostructures extending from bright seed‐like spots (Figure 4h; Figure S13). These findings demonstrate that, once incorporated into artificial LDs, 1_Phe_ undergoes seed‐initiated assembly, providing a strategy to actively regulate supramolecular growth within droplets.

## 3 Conclusion

In conclusion, we have developed lipophilic fluorescent dyes incorporating amino‐acid‐based diamides with chiral side chains, establishing a molecular design strategy for constructing supramolecular assemblies in neutral lipid droplets. By tuning the type and number of amino acid residues, fiber‐like aggregates were formed in triolein via a nucleation‐elongation mechanism. Although the fluorescence quantum yield decreased upon aggregation, the aggregated state still retained considerable luminescence, allowing direct visualization of the assemblies by fluorescence imaging. This property was attributable to the introduction of self‐assembling diamide units into the diphenylamino moiety. The aggregates displayed bisignate CD signals, demonstrating that the chirality of the two amino acid diamide residues effectively dictated the molecular orientation of the π‐conjugated core. Spectroscopic analyses revealed a kinetically trapped state that persisted for several hours, enabling real‐time monitoring of the transformation from monomers to fibrous aggregates within artificial lipid droplets. Furthermore, the moderate lipophilicity of the alkyl side chains facilitated monomer transfer from water into artificial LDs, where mixing with seed‐containing droplets modulated aggregate size, as confirmed by fluorescence imaging. These findings establish our approach as a versatile platform for programming supramolecular polymerization within neutral lipid droplets by integrating lipophilicity, hydrogen‐bonding motifs, and fluorescent functionality. We envisage that such strategies will open new opportunities at the interface between supramolecular chemistry and lipid droplet biology.

## 4 Experimental Section

### 4.1 Materials and Synthesis

All reagents and solvents used in the syntheses were purchased from commercial suppliers and used without further purification. Anhydrous CH_2_Cl_2_ was purchased from Kanto Chemicals and further purified by Glass Contour Solvent Systems. All reactions were performed under an ambient atmosphere unless stated otherwise. Thin layer chromatography (TLC) was performed on glass plates coated with 0.25 mm thickness of silica gel 60F_254_ (Merck). Column chromatography was performed using silica gel PSQ100B (Fuji Silysia Chemicals). Preparative Gel permeation Chromatography (GPC) was performed using LaboAce LC‐5060 (Japan Analytical Industry) equipped with gel column (JAIGEL‐2HR Plus) using CHCl_3_ and DMF as eluent. Preparative HPLC was performed using LaboAce LC‐5060 (Japan Analytical Industry) equipped with silica gel column (Wakosil‐II5‐Prep).

### 4.2 Characterization

^1^H and ^13^C NMR spectra were recorded with a JEOL JNM‐ECS400 spectrometer in CDCl_3_, CD_2_Cl_2_, acetone‐*d*_6_, or DMSO‐*d*_6_ (400 MHz for ^1^H, 100 MHz for ^13^C). The chemical shifts in ^1^H NMR spectra are reported in *δ* ppm using residual protons of the solvents as an internal standard (CHCl_3_ at 7.26 ppm, CH_2_Cl_2_ at 5.32 ppm, acetone at 2.05 ppm, or DMSO at 2.50 ppm). The chemical shifts in ^13^C NMR spectra are reported relative to CDCl_3_ at 77.16 ppm, CD_2_Cl_2_ at 53.84 ppm, acetone‐*d*_6_ at 29.84 or 206.26 ppm, or DMSO‐*d*_6_ at 39.52 ppm as internal standards. Mass spectra were measured with a Thermo Fisher Scientific Exactive spectrometer with the ESI ionization method. Melting points (Mp) were determined with a Yanaco MP‐S3 instrument. Full synthetic details and characterization data of new compounds can be found in the Supplementary Information.

### 4.3 Spectroscopy

Spectroscopic measurements were carried out under ambient conditions. Spectroscopic‐grade CHCl_3_, toluene, di‐*n*‐butyl ether (DBE), and DMSO were purchased from NACALAI TESQUE, INC. and used without further purification. Triolein was obtained from Tokyo Chemical Industry (TCI) and further purified by silica gel column chromatography using toluene as the eluent. UV–vis absorption spectra were recorded using quartz cuvettes of 1 cm, 2 mm, or 1 mm path length with a JASCO V‐750 spectrophotometer equipped with a JASCO ETCR‐762 cell holder for temperature control. Circular dichroism (CD) spectra were measured with a JASCO J‐1500 spectrophotometer and smoothed by savitzky‐Golay (SG) with a convolution of 25. Fluorescence spectra were recorded using JASCO FP‐8500 spectrometer.

### 4.4 TEM Studies

Transmission electron microscopy (TEM) was performed with a JEM‐1400EM (JEOL) using an acceleration voltage of 80 kV. The samples were drop‐casted on a carbon‐coat copper grid (400 mesh) and the solvent was removed with a filter paper, followed by dried under reduced pressure for 2 h.

### 4.5 Photoluminescence Quantum Yield

Absolute fluorescence quantum yields were determined using a Hamamatsu Absolute PL Quantum Yield spectrometer C11347‐01 with a calibrated integrating sphere system.

### 4.6 Confocal Laser Scanning Microscopy (CLSM) and Stimulated Emission Depletion (STED) Super‐Resolution Microscopy

Leica TCS SP8 STED 3X system including an inverted DMI6000 CS microscope equipped with a tunable (470–670 nm) pulsed white light laser (WLL; repetition rate of 78 MHz) was used. For high magnification observation, HC PL APO 63×/1.40 OIL CS2 objective was used. Images of 1_Phe_ were acquired at 470–540 nm upon excition at 405 nm. For time‐lapse observation, HC PL APO CS2 100×/1.40 oil objective was used and images of the pre‐formed 1_Phe_ seeds were obtained at 480–640 nm upon excition at 405 nm. For STED imaging, HC PL APO CS2 100×/1.40 oil objective was used and images of 1_Phe_ aggregates were obtained at 480–585 nm upon excitation at 405 nm with a 592 nm STED laser. Collected images were deconvoluted using Huygens softwere with the signal‐to‐noise ratio and quality threshold were set to 20 and 0.05 for CLSM images and 7 and 0.05 for STED images, respectively.

## Conflicts of Interest

The authors declare no conflicts of interest.

## Supporting information




**Supporting file 1**: smll72090‐sup‐0001‐SuppMat.docx.

**Supporting file 2**: smll72090‐sup‐0002‐MovieS1.mp4.

## Data Availability

The sample preparation, synthesis and characterization, supplementary figures, ^1^H and ^13^C NMR spectral data supporting this article have been included as part of the supplementary information (SI).
