# Supplementary material for: Kinetically Controlled Self‐Assembly of a Lipophilic Fluorophore with Phenylalanine‐Based Diamides in Artificial Lipid Droplets
Source: Small. 2025 Dec 29;22(10):e13411. doi: 10.1002/smll.202513411 (PMC12910420; doi:10.1002/smll.202513411)
Supplement: Supplementary file 1 — Supporting file 1: smll72090‐sup‐0001‐SuppMat.docx. [file SMLL-22-e13411-s002.docx]

Supporting Information
©Wiley-VCH 2021
69451 Weinheim, Germany

Kinetically Controlled Self-Assembly of a Lipophilic Fluorophore with Phenylalanine-based Diamides in Artificial Lipid Droplets

Miku Naruse,^[a]^ Soichiro Ogi,*^[a,b]^ Keiji Kajiwara,^[a]^ Natsumi Fukaya,^[a]^ Yoshikatsu Sato,^[c]^ Masayasu Taki,^[c,d]^ and Shigehiro Yamaguchi*^[a,b,c]^

[a] Department of Chemistry, Graduate School of Science, Nagoya University, Furo, Chikusa, Nagoya 464-8602 (Japan)
[b] Integrated Research Consortium on Chemical Science (IRCCS), Nagoya University, Furo, Chikusa, Nagoya 464-8602 (Japan)
[c] Institute of Transformative Bio-Molecules (WPI-ITbM), Nagoya University, Furo, Chikusa, Nagoya 464-8602 (Japan)
[d] Institute for Glyco-core Research (iGCORE), Gifu University, 1-1 Yanagido, Gifu 501-1193 (Japan)

ogi.soichiro@chem.nagoya-u.ac.jp, yamaguchi.shigehiro.r9@f.mail.nagoya-u.ac.jp

Table of Contents

Sample Preparation…………….……………………………………………………………………………………………....…………S3

Synthesis and Characterization…………………………………………………………….…………………………………………… S6

Supplementary Figures…………………………………………………………………………………………………..………….…… S10

References………………………………………………………………………………………………………………………………… S18

^1^H and ^13^C NMR spectra…………………………………………………………………………………………………………….…… S19

Sample Preparation

**Preparation of 1_Phe_, 1_Val_ and 1_Phe_*'*** **monomers for spectroscopic studies on the time-dependent self-assembly behavior in triolein:** A stock solution of **1_Phe_** in CHCl_3_ was transferred to vials and evaporated to dryness under reduced pressure. The residue was dissolved at 363 K in toluene at an appropriate concentration. After cooling to room temperature, triolein was then added to the resulting solution until the toluene constant reached 50 vol%, after which the toluene was removed under reduced pressure. The obtained solution was heated with a heat gun and subsequently cooled to 293 K. This solution was used for spectroscopic studies on the time-dependent self-assembly behavior. A similar procedure was employed for the preparation of compounds **1_Val_** and **1_Phe_*'*** at a concentration of 1 × 10^–5^ M.


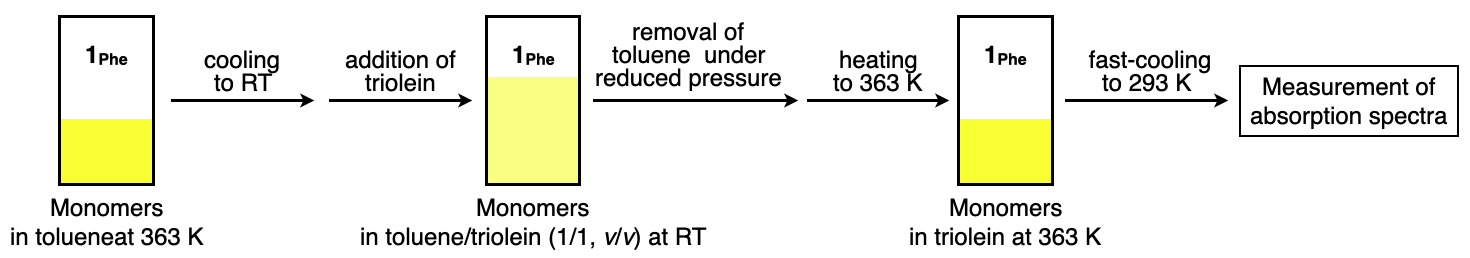


**Preparation of film sample of 1_Phe_ aggregates obtained from self-assembly in DBE:** Compound **1_Phe_** was dissolved in DBE at a concentration of 5.0 × 10^–6^ M. The resultant solution was heated with a heat gun, cooled to 293 K, and then equilibrated at 293 K for 4 h. The solution was drop-cast onto a TEM substrate (elastic carbon ELS-C10), and dried under reduced pressure for 2 h.


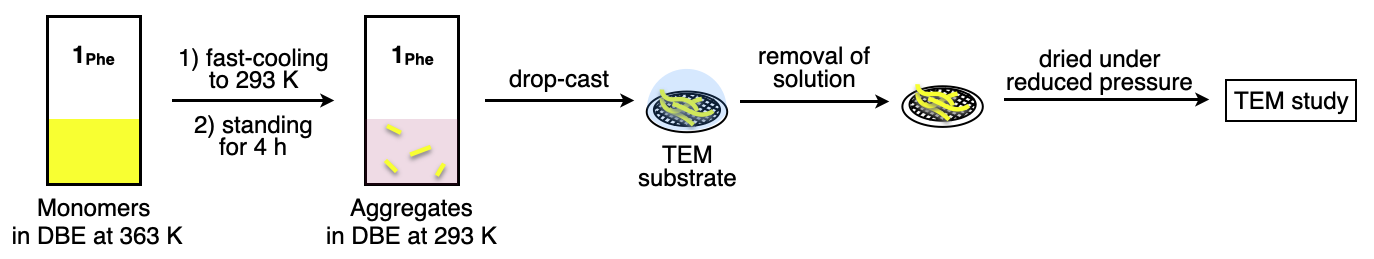


**Preparation of 1_Phe_ aggregates obtained from self-assembly in triolein for fluorescence imaging:** Compound **1_Phe_** was dissolved in triolein at a concentration of 1 × 10^–5^ M in the same manner as for the preparation of **1_Phe_** samples used in spectroscopic studies on time-dependent self-assembly behavior. The resulting solution was heated with a heat gun, cooled to 293 K, and equilibrated at this temperature for 18 h. The solution was then transferred to a glass-bottom dish and used for fluorescence imaging to observe the aggregate morphology.


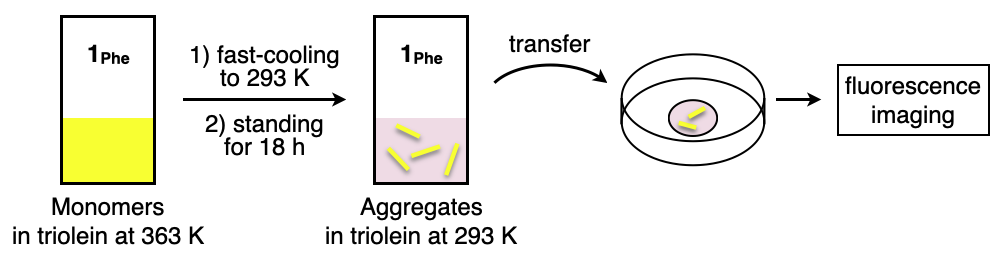


**Preparation of 1_Phe_ samples for spectroscopic studies on temperature-dependent assembly/disassembly:** Compound **1_Phe_** was dissolved in triolein at an appropriate concentration, following the procedures used for preparing samples for spectroscopic studies of time-dependent self-assembly. The resulting solution was heated with a heat gun and subsequently sonicated for 60 min in a water bath maintained at 293 K. The solution was then employed for spectroscopic measurements that follow disassembly upon heating and self-assembly upon cooling. For these temperature-dependent measurements, the sample temperature was changed at a rate of 1 K min^–1^, and at each measurement temperature the sample was allowed to equilibrate for 2 min before data acquisition.


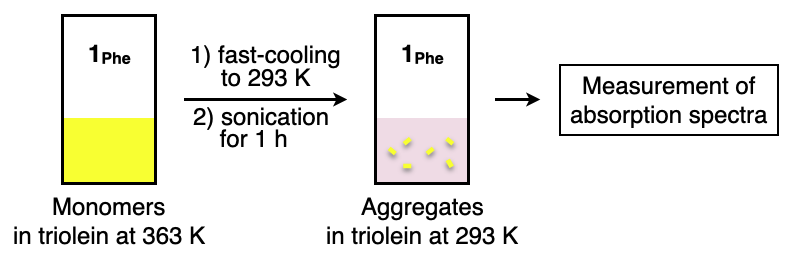


**Preparation of 1_Phe_ using the seeding method:** A seed solution of **1_Phe_** was prepared following the procedure used for spectroscopic studies on temperature-dependent self-assembly. Separately, compound **1_Phe_** was dissolved in triolein at a concentration of 1 × 10^–5^ M, as described for the preparation of samples used in time-dependent self-assembly studies. The resulting solution was heated with a heat gun, cooled at 293 K for approximately 10 min, and then placed in a cell holder maintained at 293 K. The seed solution was subsequently added to the monomeric **1_Phe_** solution, and the mixture was incubated at 293 K to induce seed-initiated assembly of **1_Phe_**.


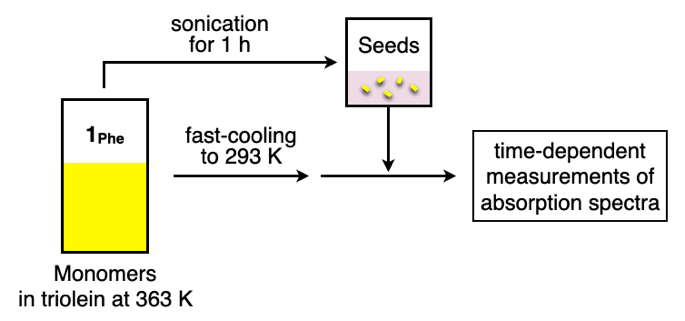


**Preparation of 1_Phe_ or 1_Val_ in artificial LDs**^S2^**:** A solution of surfactant (Brij58, 3 wt%) in distilled water was added to a solution of monomeric **1_Phe_** or **1_Val_** in triolein (at the concentrations specified in Figures S10 and S12) until the final volume fraction of the triolein solution reached 10 vol%. The mixture was then vortexed for 10 s. The resulting dispersion was used for fluorescence imaging to investigate the time-dependent self-assembly behavior.


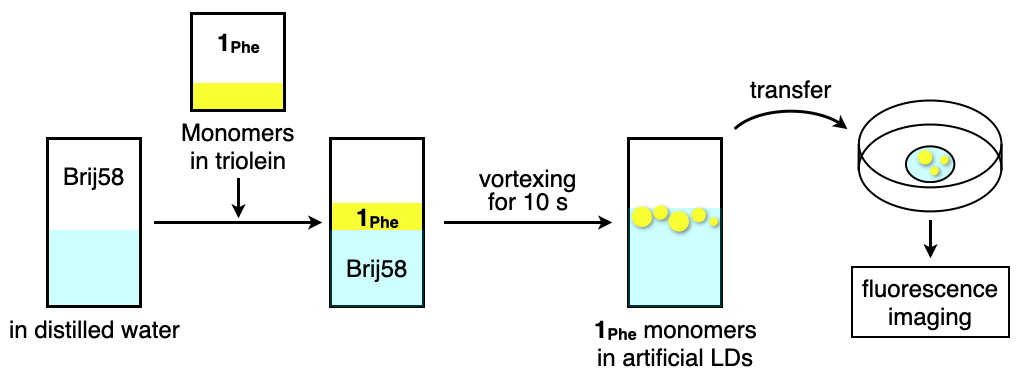


**Preparation of 1_Phe_ aggregates and Nile red in artificial LDs:** Artificial LD samples were prepared following the same procedure used for time-dependent self-assembly studies in artificial LDs, with **1_Phe_** (1 × 10^–5^ M) and Nile red (0.6 × 10^–5^ M). The resulting samples were equilibrated at 293 K for 1 d and subsequently used for fluorescence imaging of spontaneous self-assembly in artificial LDs.


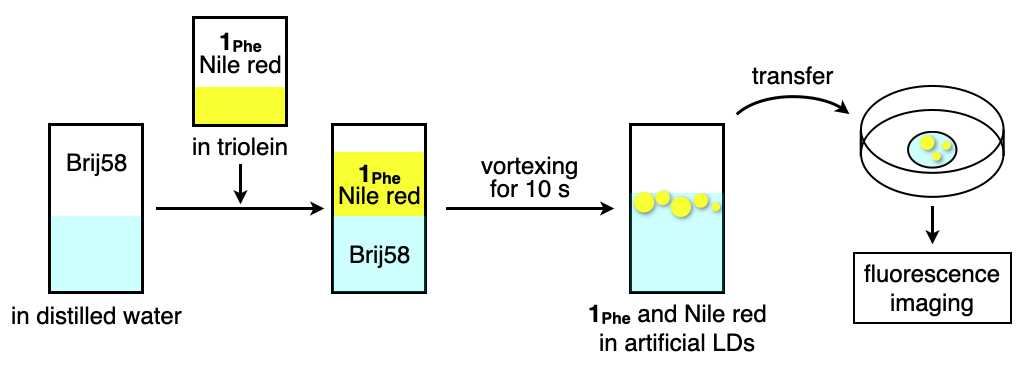


**Preparation of 1_Phe_ aggregates using a seeding method in artificial LDs:**  Artificial LD samples of **1_Phe_** were prepared following the procedure used for fluorescence imaging of self-assembly behavior in artificial LDs. A DMSO solution of **1_Phe_** at an appropriate concentration was then added until the final DMSO constant reached 10 vol%. The mixture was vortexed for an appropriate duration and subsequently equilibrated at 293 K for 2 h. The resulting samples were used for fluorescence imaging of seed-initiated assembly in artificial LDs.


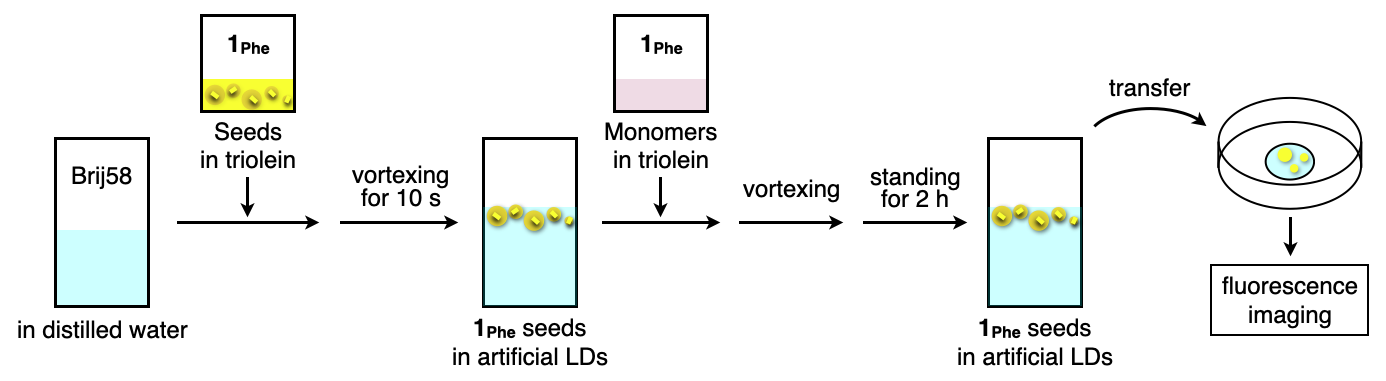


Synthesis and Characterization

**Scheme S1.** Synthesis of **1_Val_** and **1_Phe_**.

**Compound 3**. A mixture of 2-amino[1]benzothhieno[3,2-*b*][1]benzothiophene (649 mg, 2.54 mmol), CuI (169 mg, 0.887 mmol), K_2_CO_3_ (851 mg, 6.16 mmol), and ethly 4-iodobenzoate (1.30 mL, 7.82 mmol) was dissolved in degassed *o*-dichlorobenzene (25 mL) and stirred at 195 °C for 63 h under a nitrogen atmosphere. After filtration through a pad of Celite^®^, the solvent was removed under reduced pressure at around 75 °C, and the residue was dissolved in CHCl_3_. After water was added to the solution, the mixture was extracted with CHCl_3_. The combined organic layer was washed with brine, and dried over Na_2_SO_4_. After filtration, the solvent was removed under reduced pressure. The crude product was subjected to silica gel column chromatography using 3/1 hexane/EtOAc as an eluent (*R*_f_ = 0.62) to afford **3** as a white solid (939 mg, 1.70 mmol, 67%). Mp: 201.5–202.4 °C; ^1^H NMR (400 MHz, CD_2_Cl_2_): *δ* 7.97–7.85 (m, 7H), 7.70 (d, *J* = 2.0 Hz, 1H), 7.49–7.43 (m, 2H), 7.28 (dd, *J* = 8.6, 1.8 Hz, 1H), 7.18–7.14 (m, 4H), 4.33 (q, *J* = 7.2 Hz, 4H), 1.36 (t, *J* = 7.2 Hz, 6H); ^13^C NMR (100 MHz, CD_2_Cl_2_): *δ* 166.2, 151.2, 144.1, 144.0, 142.6, 133.9, 133.5, 133.4, 131.3, 130.7, 125.5, 125.4, 125.3, 124.43, 124.38, 123.0, 122.9, 121.9, 121.8, 61.1, 14.5; HRMS (ESI): *m/z* calcd. for C_32_H_25_NO_4_S_2_: 574.1117 ([*M*+Na]^+^); found: 574.1124.

**Compound 4**. A solution of **3** (210 mg, 0.381 mmol) in 1,2-dichloroethane (3.8 mL) was cooled to 0 °C, and *m*-chloroperoxybenzoic acid (*m*CPBA, 156 mg, 0.904 mmol) was added dropwise. The reaction mixture was then warmed to room temperature and stirred for 5 h. After quenching with a saturated aqueous solution of Na_2_SO_3_, the mixture was extracted with CHCl_3_ three times. The combined organic layer was washed with brine, and dried over Na_2_SO_4_. After filtration, the solvent was removed under reduced pressure. The crude product was subjected to silica gel column chromatography using CH_2_Cl_2_ as eluent (*R*_f_ = 0.40), followed by preparative HPLC using CHCl_3_ as an eluent to afford **4** as a yellow solid (79.0 mg, 0.135 mmol, 36%). Mp: 197.0–197.9 °C; ^1^H NMR (400 MHz, acetone-*d*_6_): *δ* 8.18 (d, *J* = 8.4 Hz, 1H), 8.05–8.01 (m, 4H), 7.96 (d, *J* = 8.0 Hz, 1H), 7.80 (d, *J* = 8.4 Hz, 1H), 7.66–7.61 (m, 1H) 7.59–7.54 (m, 1H), 7.47 (dd, *J* = 8.2, 2.2 Hz, 2H), 7.34–7.30 (m, 4 H), 4.35 (q, *J* = 7.1 Hz, 4H), 1.36 (t, *J* = 7.2 Hz, 6H); ^13^C NMR (100 MHz, acetone-*d*_6_): *δ* 166.1, 151.0, 149.9, 145.3, 144.4, 143.7, 134.2, 132.1, 131.1, 129.6, 127.9, 127.3, 127.2, 125.4, 125.1, 125.0, 123.6, 122.2, 118.3, 61.4, 14.6; HRMS (ESI) *m/z* calcd. for C_32_H_25_NO_6_S_2_: 606.1016 ([*M*+Na]^+^); found: 606.1017.

**Compound S2_Val_.** To a solution of (*S*)-(–)-2-methylbutylamine (0.110 mL, 0.931 mmol), *N*-(*tert*-butoxycarbonyl)-l-valine (**S1_Val_**, 245 mg, 1.13 mmol), and *O*-(benzotriazol-1-yl)-*N*,*N*,*N'*,*N'*-tetramethyluronium tetrafluoroborate (*N*-TBTU, 376 mg, 1.17 mmol) in CH_2_Cl_2_ (21 mL) was added *N*,*N*-diethylethylamine (DIEA, 0.80 mL, 4.7 mmol), and the reaction mixture was stirred at room temperature for 19 h. A saturated aqueous solution of KHSO_4_ was added to the resulting mixture, and the mixture was extracted with CH_2_Cl_2_. The combined organic layer was washed with brine, and dried over Na_2_SO_4_. After filtration, the solvent was removed under reduced pressure. The crude product was subjected to silica gel column chromatography using ethyl acetate as eluent (*R*_f_ = 0.63), followed by preparative GPC using CHCl_3_ as an eluent to afford **S2_Val_** as a white solid (243 mg, 0.848 mmol, 91%). Mp: 158.0–158.9 °C; ^1^H NMR (400 MHz, CDCl_3_): *δ* 6.47 (br, 1H), 5.29 (d, *J* = 6.4 Hz, 1H), 3.86 (dd, *J* = 8.8, 6.8 Hz, 1H), 3.13–3.19 (m, 2H), 2.04 (br, 1H), 1.60–1.25 (m, 11H), 1.17–1.02 (m, 1H), 0.96–0.77 (m, 12H); ^13^C NMR (100 MHz, acetone-*d*_6_): *δ* 172.1, 156.5, 79.0, 60.7, 45.3, 35.8, 31.9, 28.5, 27.6, 19.8, 18.1, 17.5, 11.6; HRMS (ESI): *m/z* calcd. for C_15_H_30_N_2_O_3_: 309.2149 ([*M*+Na]^+^); found: 309.2144.

**Compound S2_Phe_**. To a solution of (*S*)-(–)-2-methylbutylamine (0.160 mL, 1.35 mmol), *N*-(*tert*-butoxycarbonyl)-l-phenylalanine (**S1_Phe_**, 409 mg, 1.54 mmol), and *N*-TBTU (452 mg, 1.41 mmol) in CH_2_Cl_2_ (27 mL) was added DIEA (1.0 mL, 5.88 mmol), and the reaction mixture was stirred at room temperature for 18 h. A saturated aqueous solution of KHSO_4_ was added to the resulting mixture, and the mixture was extracted with CH_2_Cl_2_. The combined organic layer was washed with brine, and dried over Na_2_SO_4_. After filtration, the solution was removed under reduced pressure. The crude product was subjected to silica gel column chromatography using CH_2_Cl_2_ as eluent (*R*_f_ = 0.71), followed by preparative GPC using CHCl_3_ as an eluent to afford **S2_Phe_** as a white solid (427 mg, 1.28 mmol, 94%). Mp: 141.5–142.0 °C; ^1^H NMR (400 MHz, acetone-*d*_6_): *δ* 7.31–7.10 (m, 6H), 6.03 (d, *J* = 8.0 Hz, 1H), 4.31 (td, *J* = 8.4, 6.0 Hz, 1H), 3.15–2.81 (m, 4H), 1.57–1.26 (m, 11H), 1.14–0.99 (m, 1H), 0.90–0.78 (m, 6H); ^13^C NMR (100 MHz, acetone-*d*_6_): *δ* 172.0, 156.1, 138.9, 130.2, 129.0, 127.1, 79.2, 56.8, 45.4, 39.1, 35.7, 28.5, 27.4, 17.4, 11.6; HRMS (ESI): *m/z* calcd. for C_19_H_30_N_2_O_3_: 357.2149 ([*M*+Na]^+^); found: 357.2148.

**Compound 5_Val_**. To a solution of **S2_Val_** (104 mg, 0.363 mmol) in CH_2_Cl_2_ (10 mL) was added trifluoroacetic acid (TFA, 0.80 mL, 10.5 mmol), and the reaction mixture was stirred at room temperature for 14 h. After neutralization with a saturated aqueous solution of NaHCO_3_, the mixture was extracted with CH_2_Cl_2_. The combined organic layer was washed with brine, and dried over Na_2_SO_4_. After filtration, the solvent was removed under reduced pressure to afford **5_Val_** as a yellow oil, which was used in the next step without further purification.

**Compound 5_Phe_**. To a solution of **S2_Phe_** (267 mg, 0.798 mmol) in CH_2_Cl_2_ (20 mL) was added and TFA (1.7 mL, 22.2 mmol), and the reaction mixture was stirred at room temperature for 19 h. After neutralization with a saturated aqueous solution of NaHCO_3_, the mixture was extracted with CH_2_Cl_2_. The combined organic layer was washed with brine, and dried over Na_2_SO_4_. After filtration, the solvent was removed under reduced pressure to afford **5_Phe_** as a colorless oil, which was used in the next step without further purification.

**Compound 1_Val_**. To a solution of **4** (70.0 mg, 0.120 mmol) in tetrahydrofuran (THF, 3.0 mL) was added a solution of NaOH (184 mg, 4.60 mmol) in water (3.0 mL), and the reaction mixture was stirred at room temperature for 15 h. The resulting mixture was acidified with a 1 M aqueous solution of HCl and extracted with CHCl_3_ three times. The combined organic layer was washed with brine, and dried over Na_2_SO_4_. After filtration, the solvent was removed under reduced pressure. To a solution of the resulting yellow solid (53.0 mg), **5_Val_** (50 mg, 0.268 mmol), and *N*-TBTU (76.4 mg, 0.238 mmol) in DMF (3.0 mL) was added DIEA (1.0 mL, 5.9 mmol), and the reaction mixture was stirred at room temperature for 23 h. A saturated aqueous solution of KHSO_4_ and water was added to the resulting mixture. The resulting precipitate was collected by filtration and dried under reduced pressure. The crude product was subjected to silica gel column chromatography using 25/2 CHCl_3_/MeOH as an eluent (*R*_f_ = 0.38), followed by preparative GPC using DMF as an eluent to afford **1_Val_** as a yellow solid (63.5 mg, 0.0735 mmol, 61%). Mp: > 250 °C; ^1^H NMR (400 MHz, DMSO-*d*_6_): *δ* 8.29–8.18 (m, 3H), 8.03–7.87 (m, 7H), 7.85 (d, *J* = 8.4 Hz, 1H), 7.64–7.50 (m, 2H), 7.45 (d, *J* = 2.0 Hz, 1H), 7.32 (dd, *J* = 8.0, 1.6 Hz, 1H), 7.22 (d, *J* = 8.0 Hz, 4H), 4,34–4.23 (m, 2H), 3.00–2.91 (m, 4H), 2.17–2.03 (m, 2H), 1.57–1.43 (m, 2H), 1.43–1.29 (m, 2H), 1.14–1.00 (m, 2H), 0.96–0.78 (m, 24H); ^13^C NMR (400 MHz, DMSO-*d*_6_): *δ* 171.0, 165.5, 149.1, 148.1, 144.0, 143.4, 142.3, 131.7, 130.3, 129.6, 129.5, 127.21, 127.17, 126.4, 125.0, 124.7, 124.2, 121.0, 115.7, 59.2, 44.2, 34.3, 30.1, 26.5, 19.4, 18.9, 17.2, 11.2; HRMS (ESI): *m/z* calcd. for C_48_H_57_N_5_O_6_S_2_: 862.3678 ([*M*–H]^–^); found: 862.3663.

**Compound 1_Phe_**. To a solution of **4** (190 mg, 0.326 mmol) in THF (6.0 mL) was added a solution of NaOH (275 mg, 6.88 mmol) in water (6.0 mL), and the reaction mixture was stirred at room temperature for 21 h. The resulting mixture was acidified with a 1 M aqueous solution of HCl and extracted with 1/1 CHCl_3_/acetone three times. The combined organic layer was washed with brine, and dried over Na_2_SO_4_. After filtration, the solvent was removed under reduced pressure. To a solution of the resulting yellow solid (137 mg), **5_Phe_** (198 mg, 0.845 mmol), and *N*-TBTU (203 mg, 0.632 mmol) in DMF (8.0 mL) was added DIEA (0.600 mL, 3.53 mmol), and the reaction mixture was stirred at room temperature for 23 h. A saturated aqueous solution of KHSO_4_ and water was added to the resulting mixture. The resulting precipitate was collected by filtration and dried under reduced pressure. The crude product was subjected to silica gel column chromatography using 25/2 CHCl_3_/MeOH as an eluent (*R*_f_ = 0.48), followed by preparative GPC using DMF as an eluent to afford **1_Phe_** as a yellow solid (155 mg, 0.161 mmol, 50%). Mp: 246.5–247.0 °C; ^1^H NMR (400 MHz, DMSO-*d*_6_): *δ* 8.57 (d, *J* = 8.8 Hz, 1H), 8.25 (d, *J* = 8.0 Hz, 1H), 8.04 (t, *J* = 5.6 Hz, 1H), 7.90 (d, *J* = 7.6 Hz, 1H), 7.87–7.78 (m, 5H), 7.66–7.48 (m, 2H), 7.41 (d, *J* = 2.4 Hz, 1H), 7.37–7.10 (m, 15H), 4.76–4.64 (m, 2H), 3.13–2.85 (m, 8H), 1.55–1.25 (m, 4H), 1.11–0.96 (m, 2H), 0.90–0.74 (m, 12H); ^13^C NMR (400 MHz, DMSO-*d*_6_): *δ* 171.33, 171.26, 165.4, 165.3, 149.1, 144.1, 143.4, 142.3, 148.1, 131.7, 130.03, 129.6, 138.5, 129.5, 129.2, 128.1, 127.3, 127.2, 126.4, 126.3, 125.0, 124.7, 124.2, 121.05, 121.01, 115.9, 55.1, 44.3, 44.2, 37.5, 34.5, 26.4, 17.2, 11.3; HRMS (ESI): *m/z* calcd. for C_56_H_57_N_5_O_6_S_2_: 982.3642 ([*M*+Na]^+^); found: 982.3656.

**Scheme S2.** Synthesis of **1_Phe_*'***.

**Compound 3*'***. A mixture of 2-amino[1]benzothhieno[3,2-*b*][1]benzothiophene (58.0 mg, 0.227 mmol), iodobenzene (78.2 mg, 0.383 mmol), CuI (15.3 mg, 80.3 μmol), K_2_CO_3_ (145 mg, 1.05 mmol), and ethyl 4-iodobenzoate (112 mg, 0.406 mmol) was dissolved in degassed *o*-dichlorobenzene (1.2 mL) and stirred at 195 °C for 36 h under a nitrogen atmosphere. After filtration through a pad of Celite^®^, the solvent was removed under reduced pressure. The crude product was subjected to silica gel column chromatography using 4/1 hexane/CH_2_Cl_2_ as eluent (*R*_f_ = 0.15) to afford **3*'*** as a white solid (30.8 mg, 64.2 μmol, 28%). Mp: 102.0–102.9 °C; ^1^H NMR (400 MHz, acetone-*d*_6_): *δ* 8.09 (d, *J* = 8.0 Hz, 1H), 7.97 (dd, *J* = 8.8, 1.2 Hz, 2H), 7.90 (d, *J* = 8.8 Hz, 2H), 7.83 (d, *J* = 1.6 Hz, 1H), 7.57–7.45 (m, 2H), 7.42 (t, *J* = 7.8 Hz, 2H), 7.35–7.29 (m, 1H), 7.27–7.18 (m, 3H), 7.07 (d, *J* = 8.8 Hz, 2H), 4.31 (q, *J* = 7.2 Hz, 2H), 1.34 (t, *J* = 7.2 Hz, 3H); ^13^C NMR (100 MHz, acetone-*d*_6_): *δ* 166.3, 152.8, 149.5, 145.4, 144.5, 142.9, 133.9, 133.8, 131.6, 130.7, 130.5, 126.9, 126.1, 125.8, 125.1, 124.6, 123.9, 123.3, 122.2, 121.6, 121.1, 60.1, 14.7; HRMS (ESI): *m/z* calcd. for C_29_H_21_NO_2_S_2_: 502.0906 ([*M*+Na]^+^); found: 502.0906.

**Compound 4*'***. A solution of **3*'*** (203 mg, 0.423 mmol) in 1,2-dichloroethane (5.0 mL) was cooled to 0 °C, and *m*CPBA (172 mg, 0.997 mmol) was added dropwise. The reaction mixture was then warmed to room temperature and stirred for 5 h. The quenching with a saturated aqueous solution of Na_2_SO_3_, the mixture was extracted with CHCl_3_ three times. The combined organic layer was washed with brine, and dried over Na_2_SO_4_. After filtration, solvent was removed under reduced pressure. The crude product was subjected to silica gel column chromatography using 1/1 hexane/CH_2_Cl_2_ as eluent (*R*_f_ = 0.45), followed by preparative HPLC to afford **4*'*** as a yellow solid (56.2 mg, 0.110 mmol, 26%). Mp: 126.0–126.9 °C; ^1^H NMR (400 MHz, acetone-*d*_6_): *δ* 8.16 (d, *J* = 8.4 Hz, 1H), 8.01–7.97 (m, 2 H), 7.94 (d, *J* = 8.4 Hz, 1H), 7.74 (d, *J* = 8.4 Hz, 1H), 7.65–7.60 (m, 1H), 7.57–7.47 (m. 3H), 7.43 (d, *J* = 2.0 Hz, 1H), 7.37 (dd, *J* = 8.4, 2.4 Hz, 1H), 7.34–7.29 (m, 3H), 7.26–7.21 (m, 2H), 4.34 (q, *J* = 7.2 Hz, 2H), 1.35 (t, *J* = 7.2 Hz, 3H); ^13^C NMR (100 MHz, acetone-*d*_6_): 166.1, 151.5, 150.6, 146.6, 145.2, 144.7, 143.5, 133.8, 131.9, 131.22, 131.17, 127.8, 127.70, 127.66, 127.2, 127.0, 126.1, 125.3, 124.8, 123.7, 122.2, 122.1, 116.5, 61.3, 14.6; HRMS (ESI): *m/z* calcd. for C_29_H_21_NO_4_S_2_: 534.0804 ([*M*+Na]^+^); found: 534.0797.

**Compound 1_Phe_*'***. To a solution of **4*'*** (51.2 mg, 0.100 mmol) in THF (3.0 mL) was added a solution of NaOH (190 mg, 4.75 mmol) in water (3.0 mL), and the reaction mixture was stirred at room temperature for 23 h. The resulting mixture was acidified with 1 M aqueous solution of HCl and extracted with 1/1 CHCl_3_/acetone. The combined organic layer was washed with brine, and dried over Na_2_SO_4_. After filtration, the solvent was removed under reduced pressure. To a solution of the resulting yellow oil matter (52.0 mg), **5_Phe_** (54.1 mg, 0.231 mmol), and *N*-TBTU (88.9 mg, 0.277 mmol) in DMF (2.7 mL) was added DIEA (0.200 mL, 1.18 mmol), and the reaction mixture was stirred at room temperature for 118 h. A saturated aqueous solution of KHSO_4_ and water was added to the resulting mixture. The resulting precipitate was collected by filtration and dried under reduced pressure. The crude product was subjected to silica gel column chromatography using 25/1 CHCl_3_/MeOH as an eluent (*R*_f_ = 0.38), followed by preparative GPC using DMF as an eluent to afford **1_Phe_*'*** as a yellow solid (63.4 mg, 0.0906 mmol, 91%). Mp: 146.5–147.0 °C; ^1^H NMR (400 MHz, DMSO-*d*_6_): *δ* 8.52 (d, *J* = 8.8 Hz, 1H), 8.23 (d, *J* = 8.0 Hz, 1H), 8.00 (t, *J* = 6.0 Hz, 1H), 7.88 (d, *J* = 8.0 Hz, 1H), 7.84–7.79 (m, 3H), 7.61–7.43 (m, 4H), 7.35–7.14 (m, 12H) 4.73–4.66 (m, 1H), 3.10–2.89 (m, 4H), 1.52–1.26 (m, 2H), 1.10–0.98 (m, 1H), 0.86–0.78 (m, 6H); ^13^C NMR (100 MHz, DMSO-*d*_6_): *δ* 171.3, 165.4, 149.6, 148.4, 145.3, 144.2, 143.4, 142.2, 138.5, 131.4, 130.3, 129.62, 129.57, 129.3, 129.1, 128.1, 127.1, 126.3, 126.2, 125.9, 125.6, 124.9, 124.6, 123.5, 120.9, 119.9, 114.1, 55.0, 44.3, 37.5, 34.4, 26.4, 17.1, 11.2; HRMS (ESI): *m/z* calcd. for C_41_H_37_N_3_O_4_S_2_: 722.2118 ([*M*+Na]^+^); found: 722.2138.

Supplementary Figures


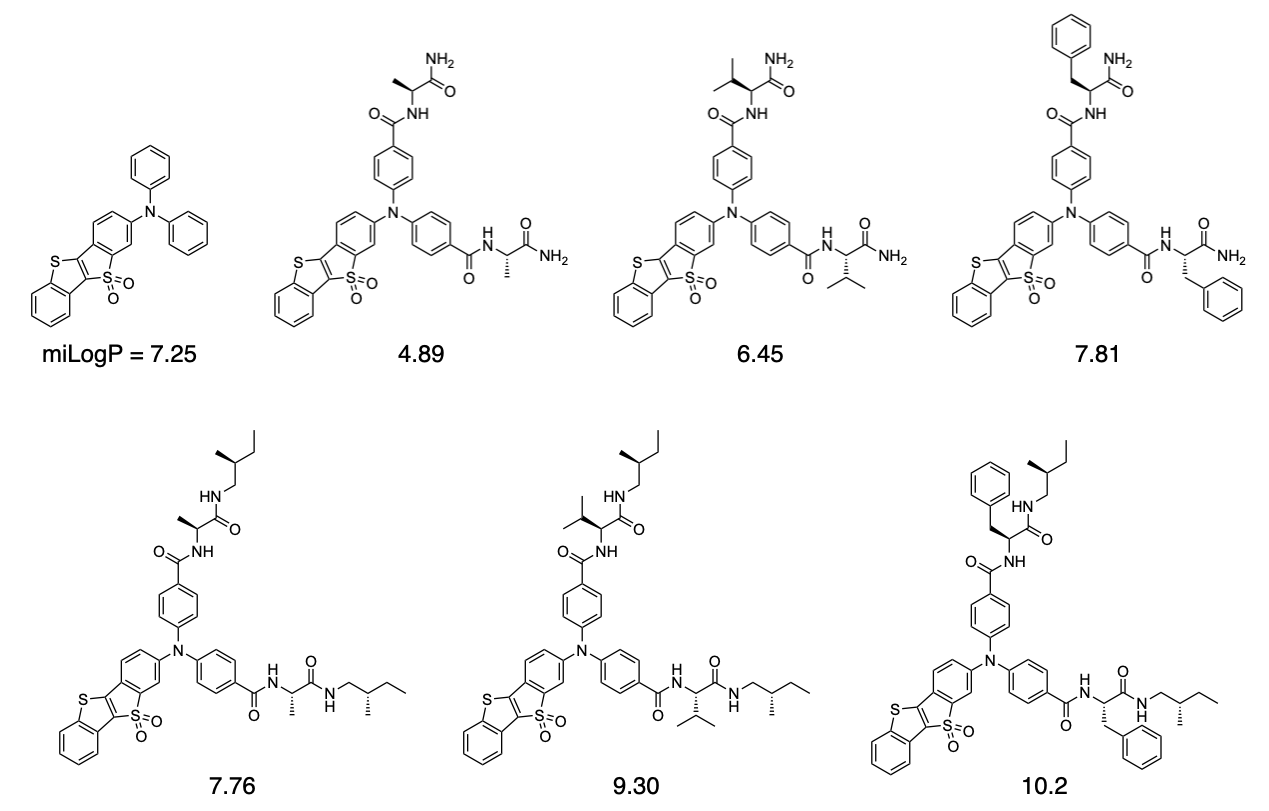


**Figure S1.** Chemical structures of LAQ derivatives obtained by introducing the diamide units shown into the diphenylamino group of **LAQ1** and their miLogP values calculated with Molinspiration software.^S3^


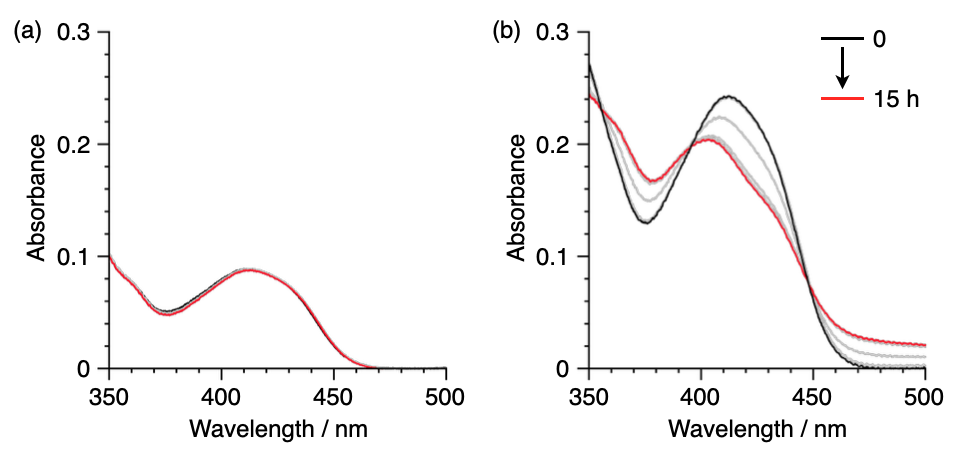


**Figure S2.** The time-dependent UV-vis absorption spectra of **1_Phe_** in triolein at a concentration of (a) 5.0 × 10^–6^ M and (b) 1.5 × 10^–5^ M.


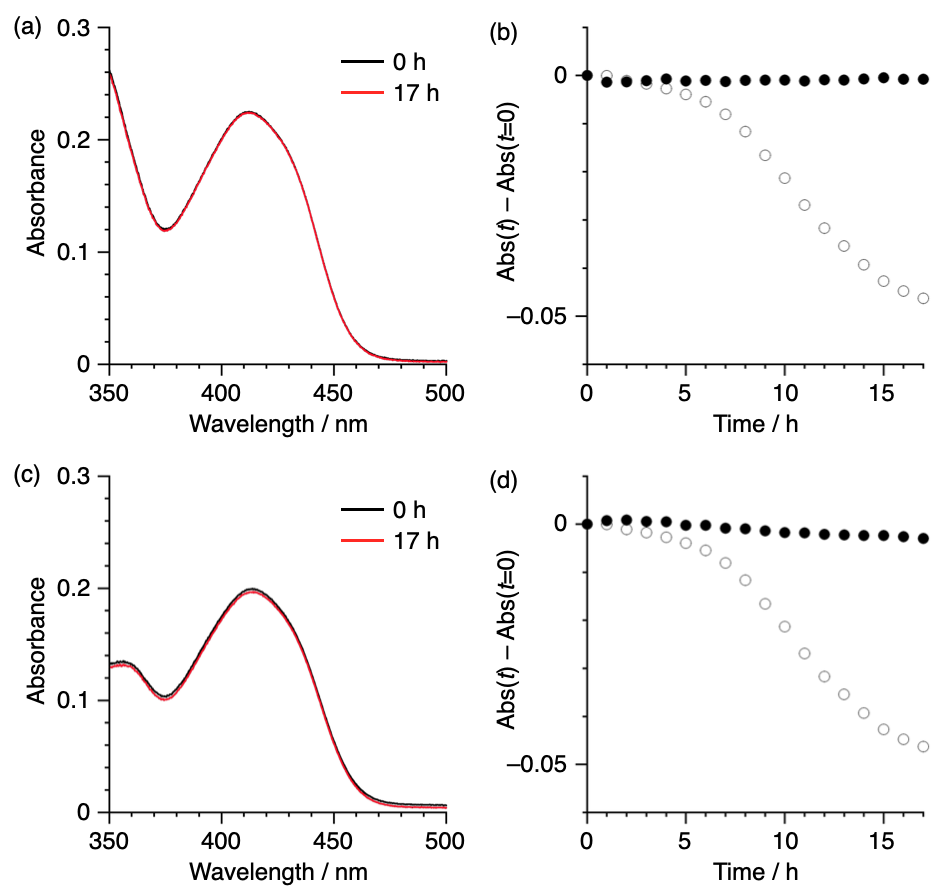


**Figure S3.** Time-dependent changes of UV-vis absorption spectra for (a) **1_Val_** in triolein at a concentration of 1.1 ×10^–4^ M and (c) **1_Phe_*'*** in triolein at a concentration of 1.0 ×10^–5^ M, measured immediately after cooling a hot solution to 293 K (black line) and after 17 hours (red line). Spectra were recorded using quartz cuvettes of with a path length of 1 mm for the **1_Val_** sample and 1 cm for the **1_Phe_*'*** sample. (b,d) Time courses of the absorbance at 411 nm for (b) **1_Val_** and (d) **1_Phe_*'*** (closed circles), shown together with the corresponding data for **1_Phe_** (open circles; 1.0 × 10^–5^ M) for comparison.


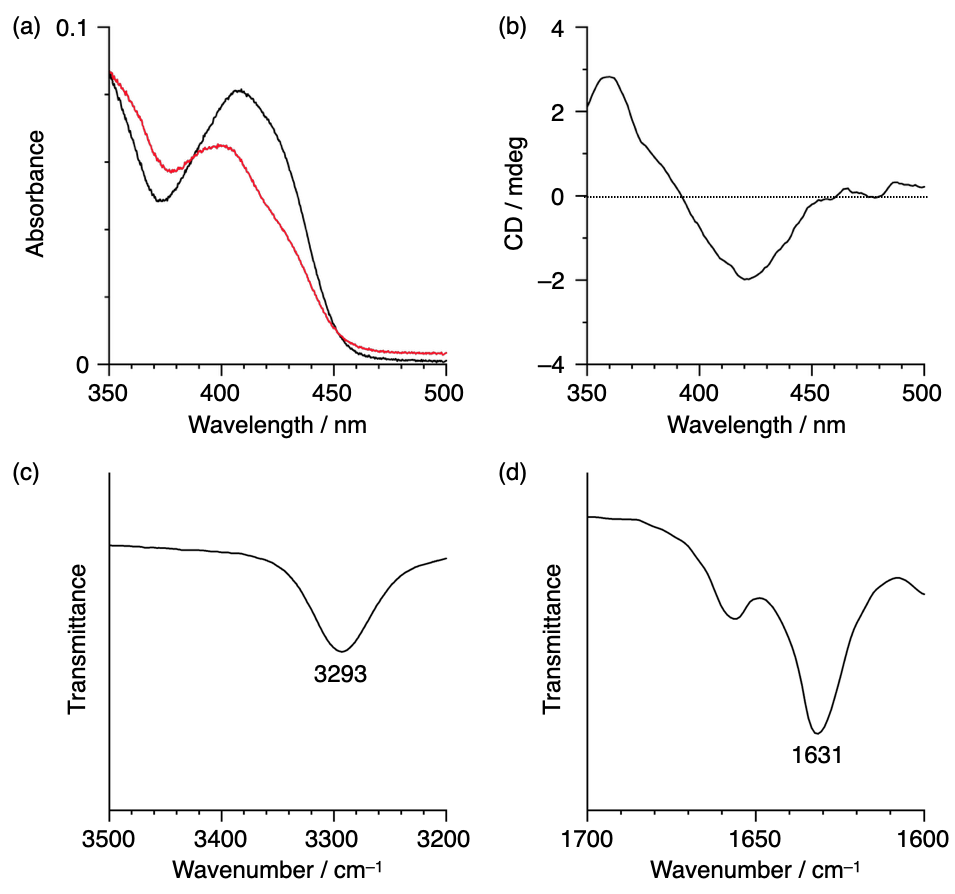


**Figure S4.** (a) The UV-vis absorption spectra of **1_Phe_** in di-*n*-butyl ether (DBE) at a concentration of 5.0 ×10^–6^ M before sonication (black line) and after sonication (red line). (b) CD spectrum of the sonicated sample. (c,d) FT-IR spectra of (c) N–H and (d) C=O stretching bands for a thin film of **1_Phe_** aggregates obtained by drop-casting a 5.0 × 10^–6^ M solution in DBE.


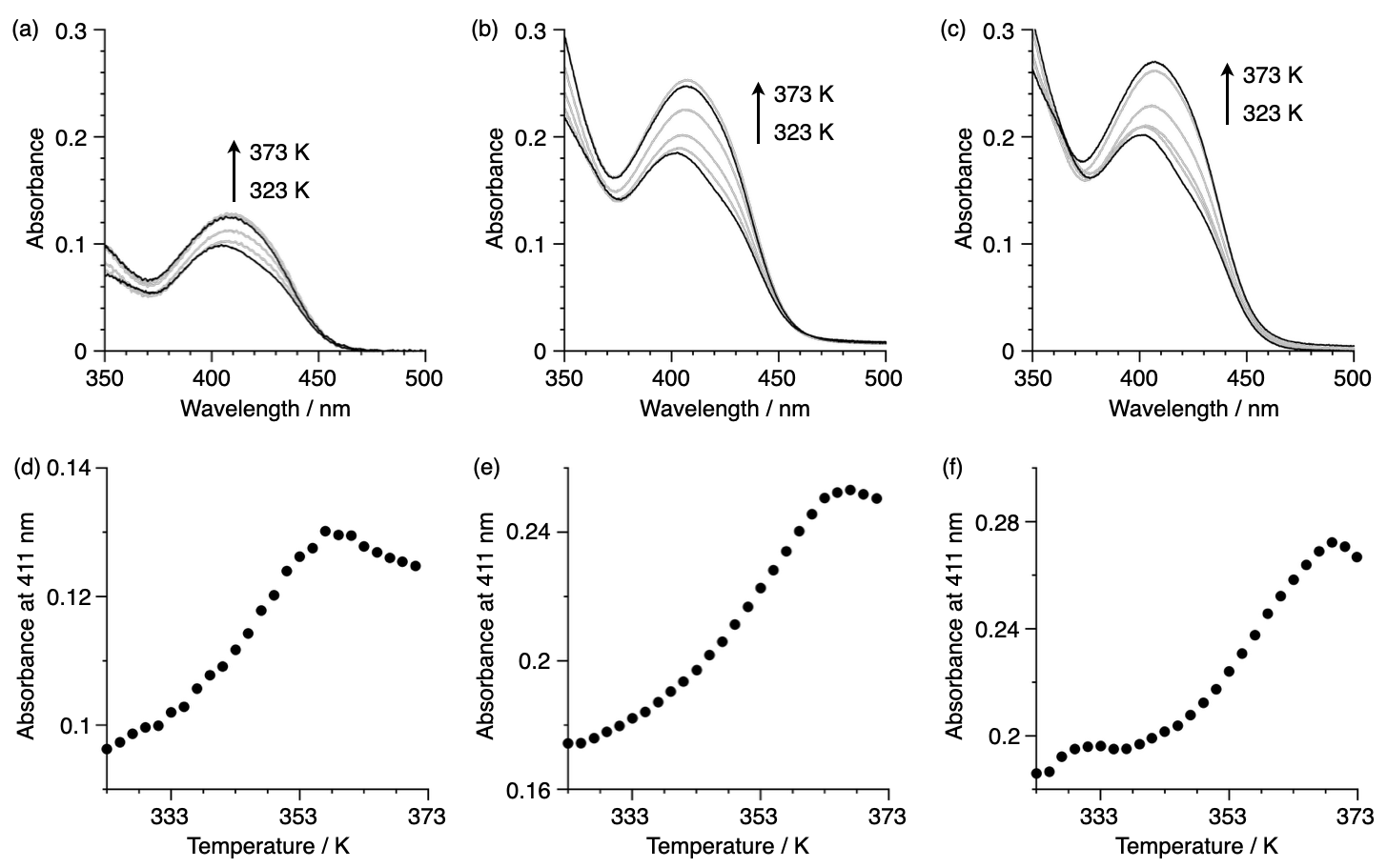


**Figure S5.** (a–c) UV/vis absorption spectral changes of **1_Phe_** in triolein at 7.5 × 10^–6^ M (a), 1.3 × 10^–5^ M (b), 1.5 × 10^–5^ M (c) upon increasing temperature form 317 K to 373 K at a rate of 1 K min^–1^, and (d–f) the respective temperature-dependent changes in absorbance at 411 nm.


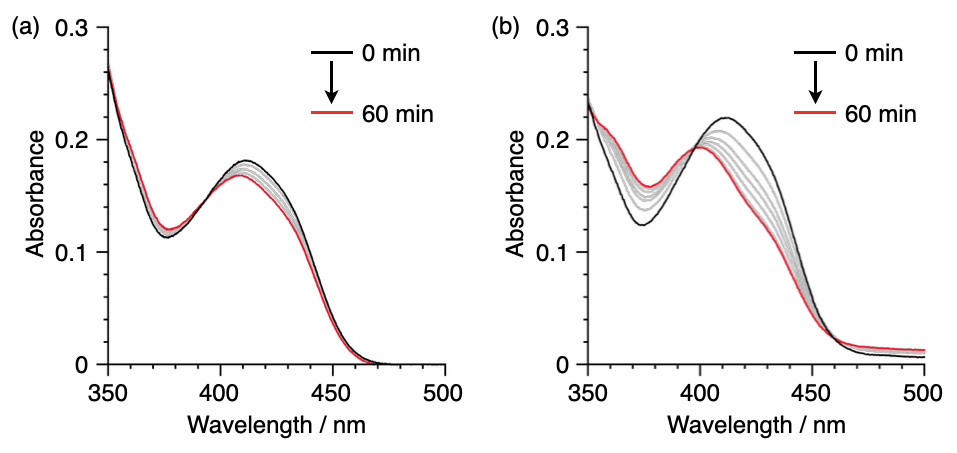


**Figure S6.** (a,b) Time-dependent changes in the absorption spectra of **1_Phe_** in triolein before and after addition of a solution of **1_Phe_** seeds in triolein (0.6 mL for a; 0.2 mL for b; 1.0 × 10^–5^ M) to **1_Phe_** monomers in triolein (2.4 mL for a; 2.3 mL for b; 1.0 × 10^–5^ M).


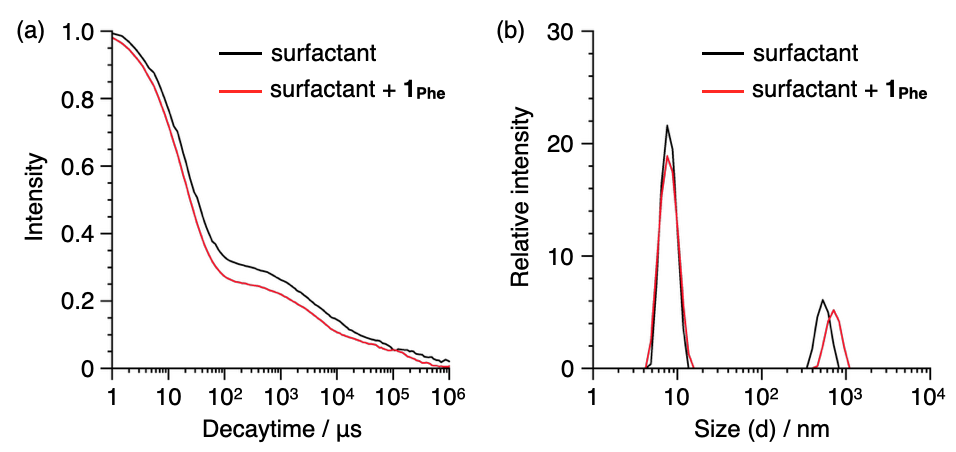


**Figure S7.** (a) DLS correlation function and (b) intensity size distribution; black lines: a solution of Brij58 surfactant (3 wt%) in water; red lines: a mixture of **1_Phe_** solution in DMSO (1.0 × 10^–4^ M; 0.28 mL) and surfactant solution in water (3 wt%; 2.8 mL).


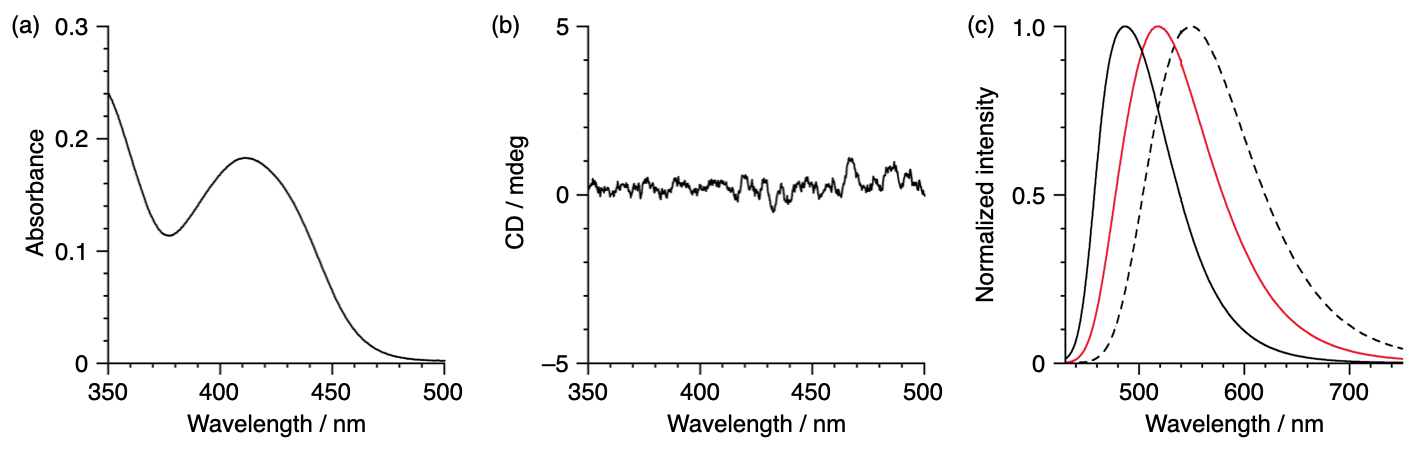


**Figure S8.** (a) UV-vis absorption and (b) CD spectra of a mixture of **1_Phe_** in DMSO (1.0 × 10^–4^ M; 0.28 mL) and Brij58 surfactant in water (3 wt%; 2.8 mL). (c) Fluorescence spectra of **1_Phe_** monomers in triolein (1.0 × 10^–5^ M; solid black line), **1_Phe_** monomers in DMSO (1.0 × 10^–5^ M; dashed black line), and the mixture of **1_Phe_** in DMSO (1.0 × 10^–4^ M; 0.28 mL) with Brij58 surfactant in water (3 wt%; 2.8 mL) (red line).


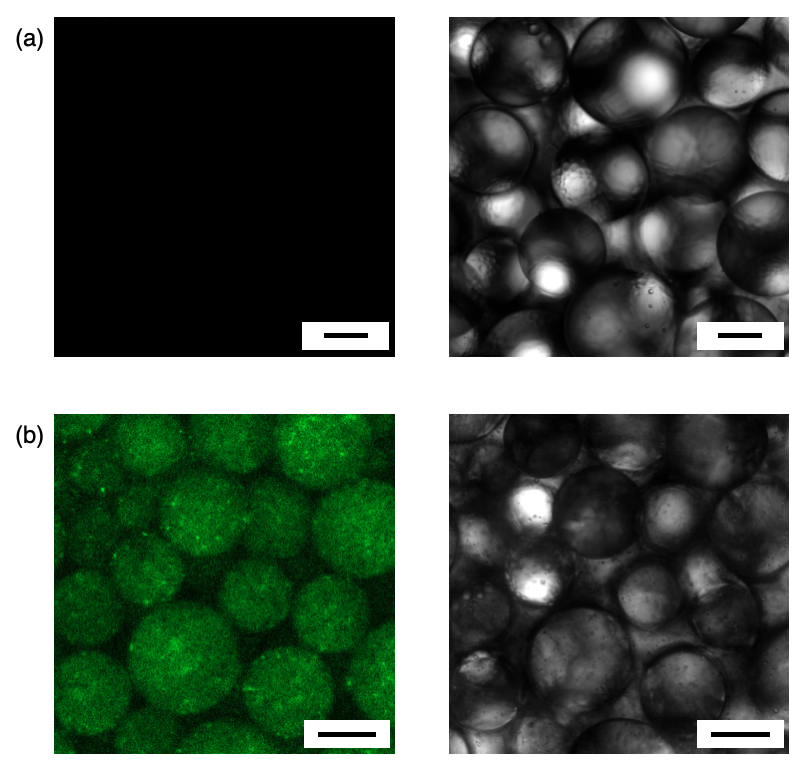


**Figure S9.** CLSM (left) and bright (right) images of (a) artificial LDs prepared without **1_Phe_** and (b) droplets obtained after addition of **1_Phe_** solution in DMSO, vortexed for 210 s, followed by standing for 2 h; *λ*_ex_ = 405 nm, *λ*_em_ = 470–540 nm; scale bars: 50 µm.


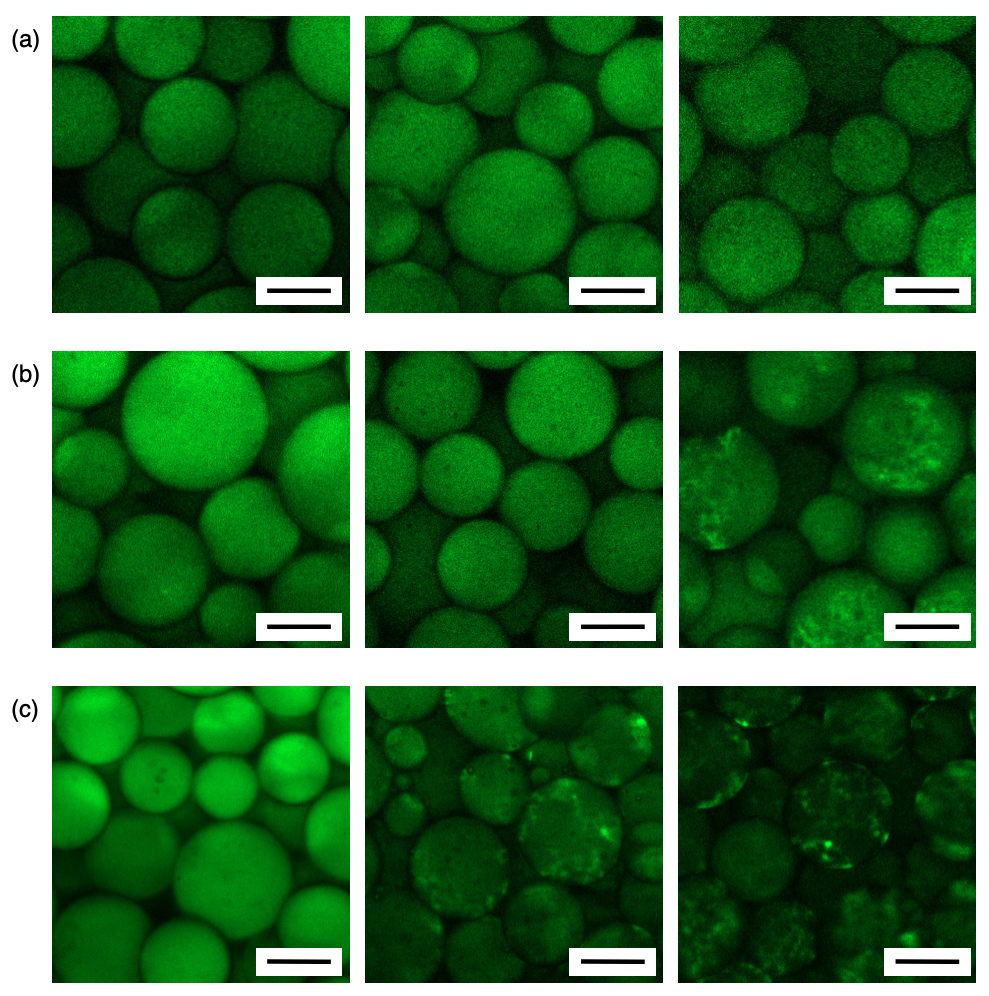


**Figure S10.** CLSM images of artificial LDs containing **1_Phe_** at concentrations of (a) 5.0 × 10^–6^ M, (b) 1.0 × 10^–5^ M, and (c) 1.5 × 10^–5^ M observed just after (left), 4 h after (middle), and 1 d after (right) the sample preparation; *λ*_ex_ = 405 nm, *λ*_em_ = 470–540 nm; scale bars: 50 µm.


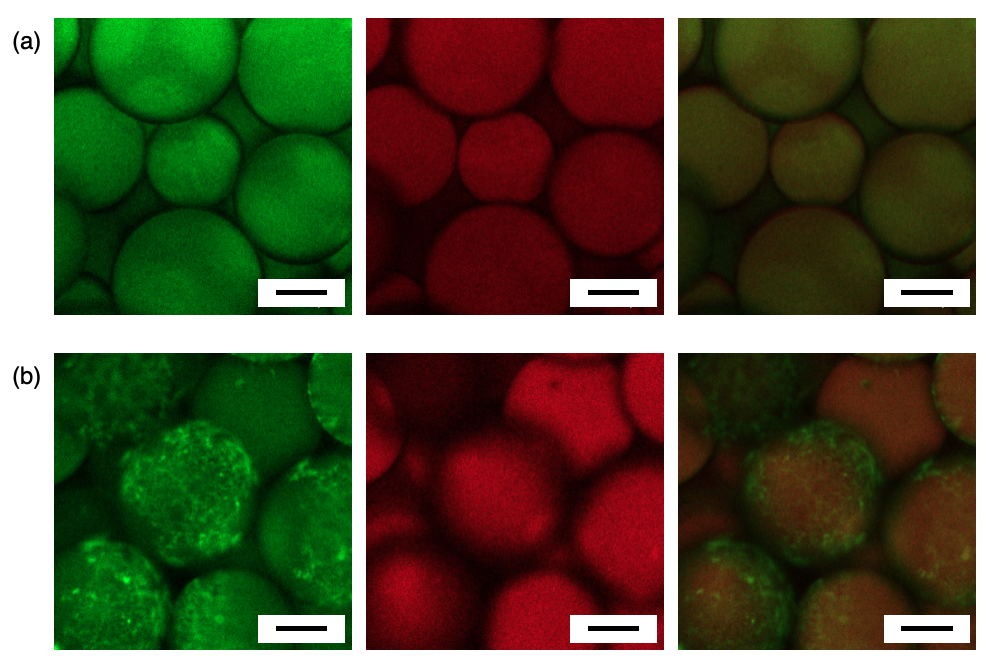


**Figure S11.** CLSM images of **1_Phe_** aggregates (1.0 × 10^–5^ M) with Nile red (6.7 × 10^–6^ M) in artificial LDs just after (a) and 1 d after (b) the sample preparation; left (green channel): *λ*_ex_ = 405 nm, *λ*_em_ = 470–540 nm; middle (red channel): *λ*_ex_ = 556 nm, *λ*_em_ = 650–695 nm; right: overlay images; scale bars: 50 µm.


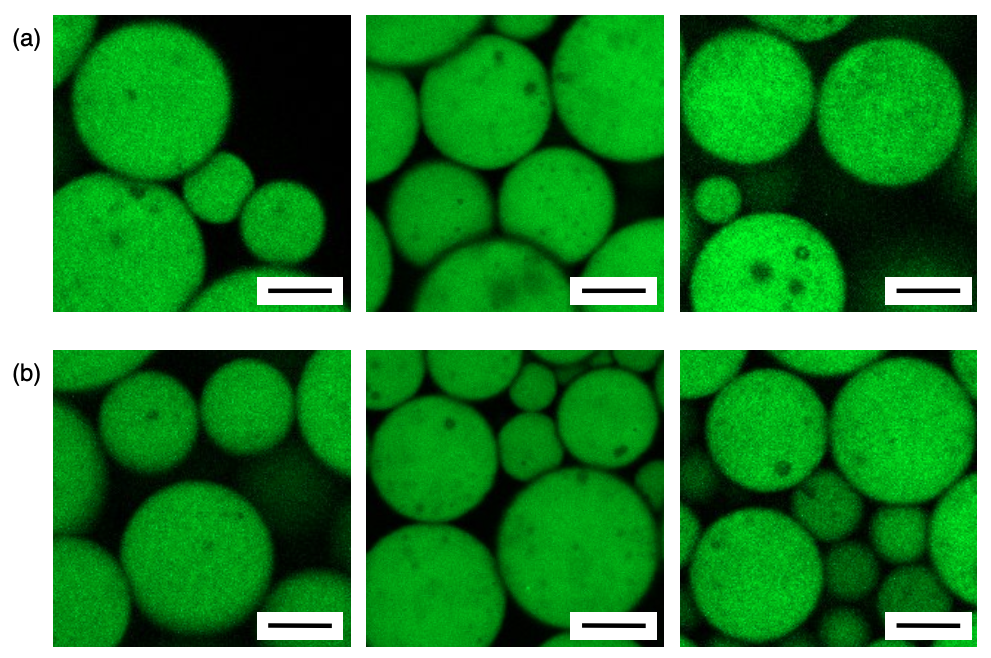


**Figure S12.** CLSM images of artificial LDs containing **1_Val_** at concentrations of (a) 1.0 × 10^–5^ M and (b) 1.5 × 10^–5^ M observed just after (left), 4 h after (middle), and 1 d after (right) the sample preparation; *λ*_ex_ = 405 nm, *λ*_em_ = 470–540 nm; scale bars: 50 µm.


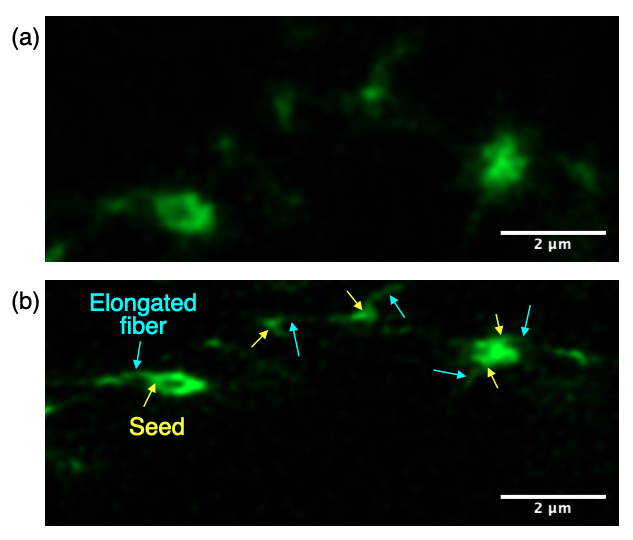


**Figure S13.** (a) CLSM image of **1_Phe_** aggregates obtained after adding monomeric **1_Phe_** in DMSO to water containing artificial LDs with pre-formed **1_Phe_** seeds; *λ*_ex_ = 405 nm, *λ*_em_ = 480–640 nm., and (b) corresponding STED image of the same region shown in (a); λ_ex_ = 405 nm, λ_STED_ = 592 nm, λ_em_ = 480–585nm.

# References

[S1] B. Košata, V. Kozmík, J. Svoboda, *Collect. Czech. Chem. Commun*. **2002**, *67*, 645–664.

[S2] S. F. Shimobayashi, Y. Ohsaki, *Proc. Natl. Acad. Sci. USA* **2019**, *116*, 25440–25445.

[S3] R. Mannhold, G. I. Poda, C. Ostermann, I. V. Tetko, *J. Pharm. Sci*, **2009**, *98*, 861–893.

**^1^H and ^13^C NMR spectra**


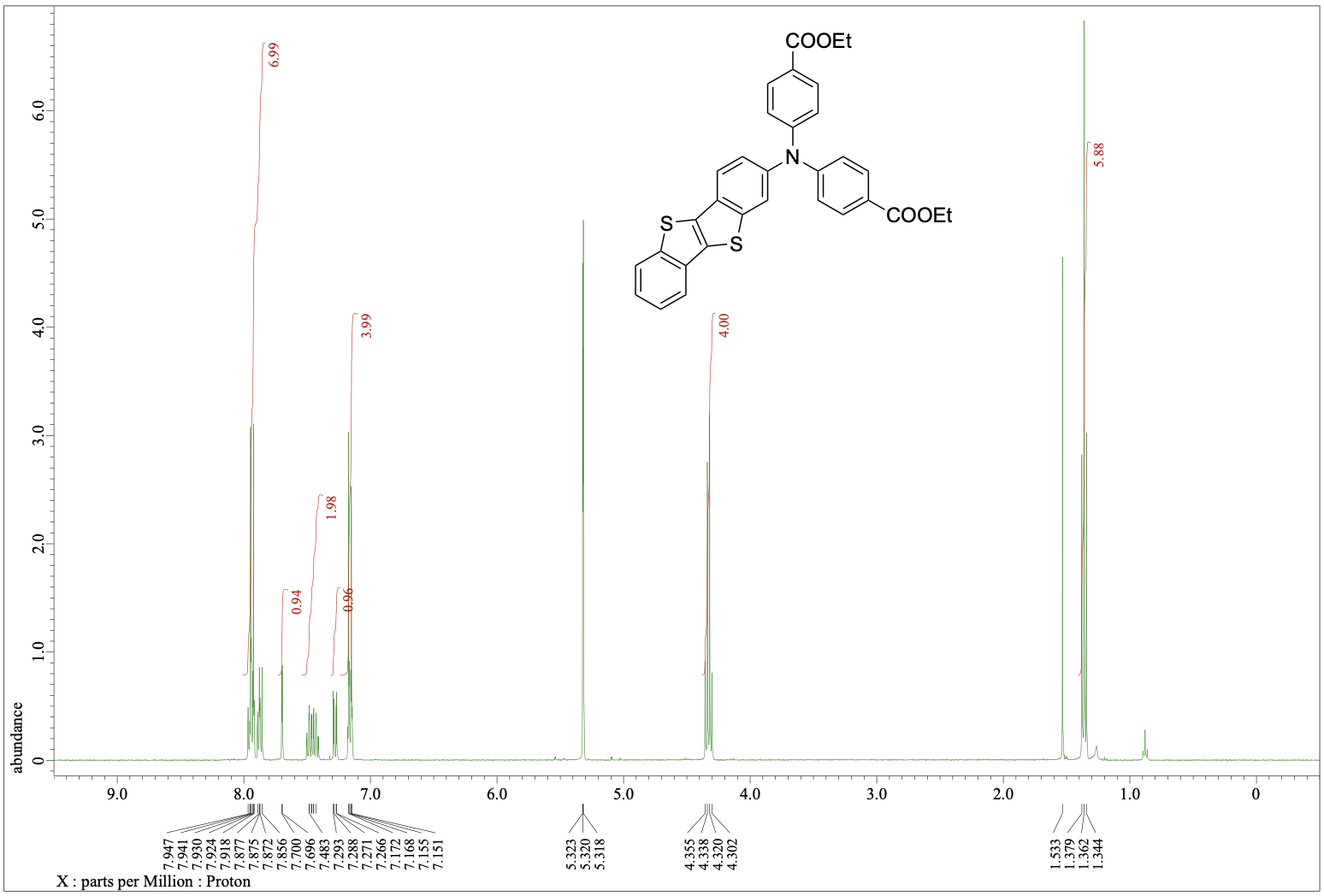


**Figure S14**. ^1^H NMR spectrum of **3** (400 MHz, CD_2_Cl_2_).


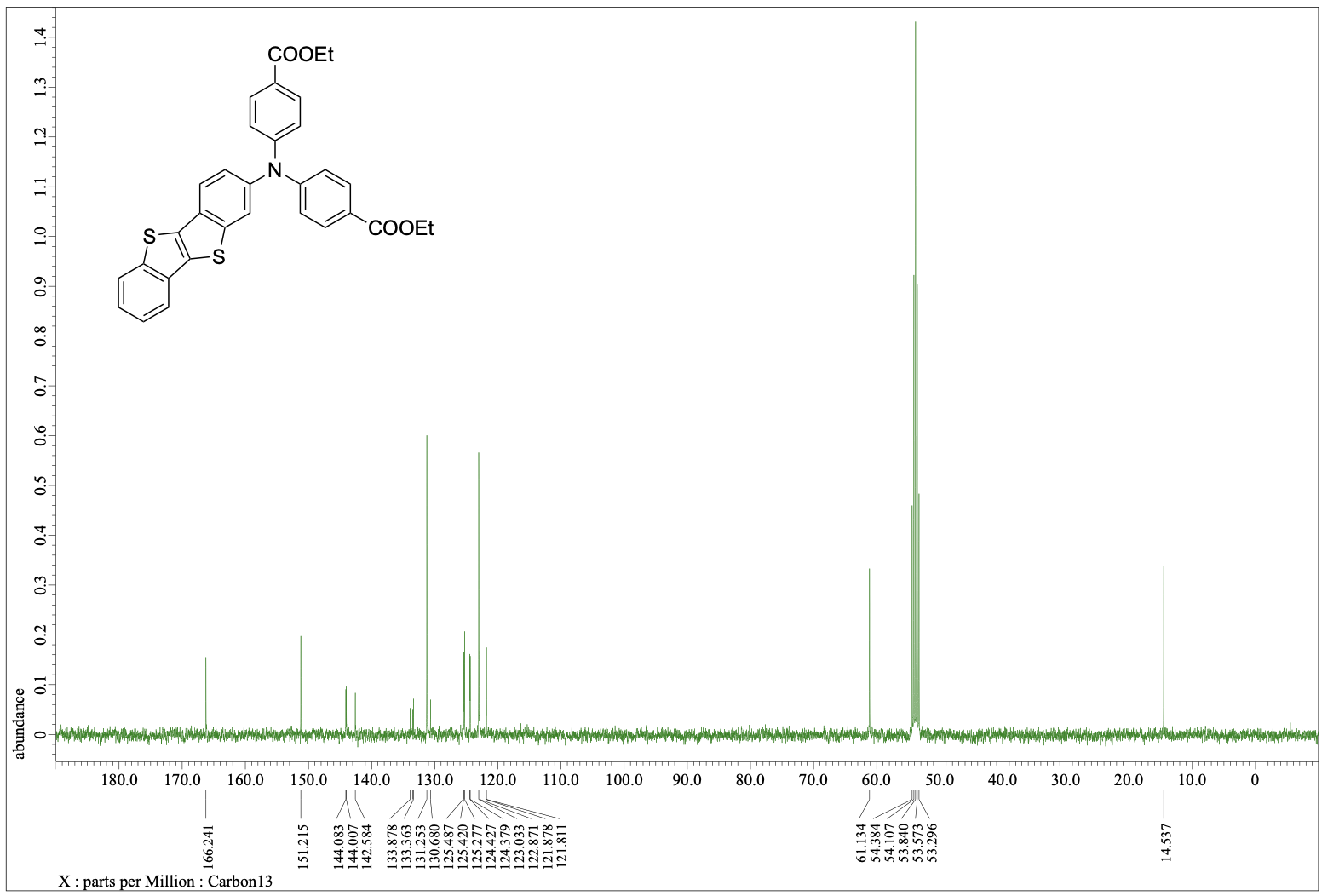


**Figure S15**. ^13^C NMR spectrum of **3** (100 MHz, CD_2_Cl_2_).


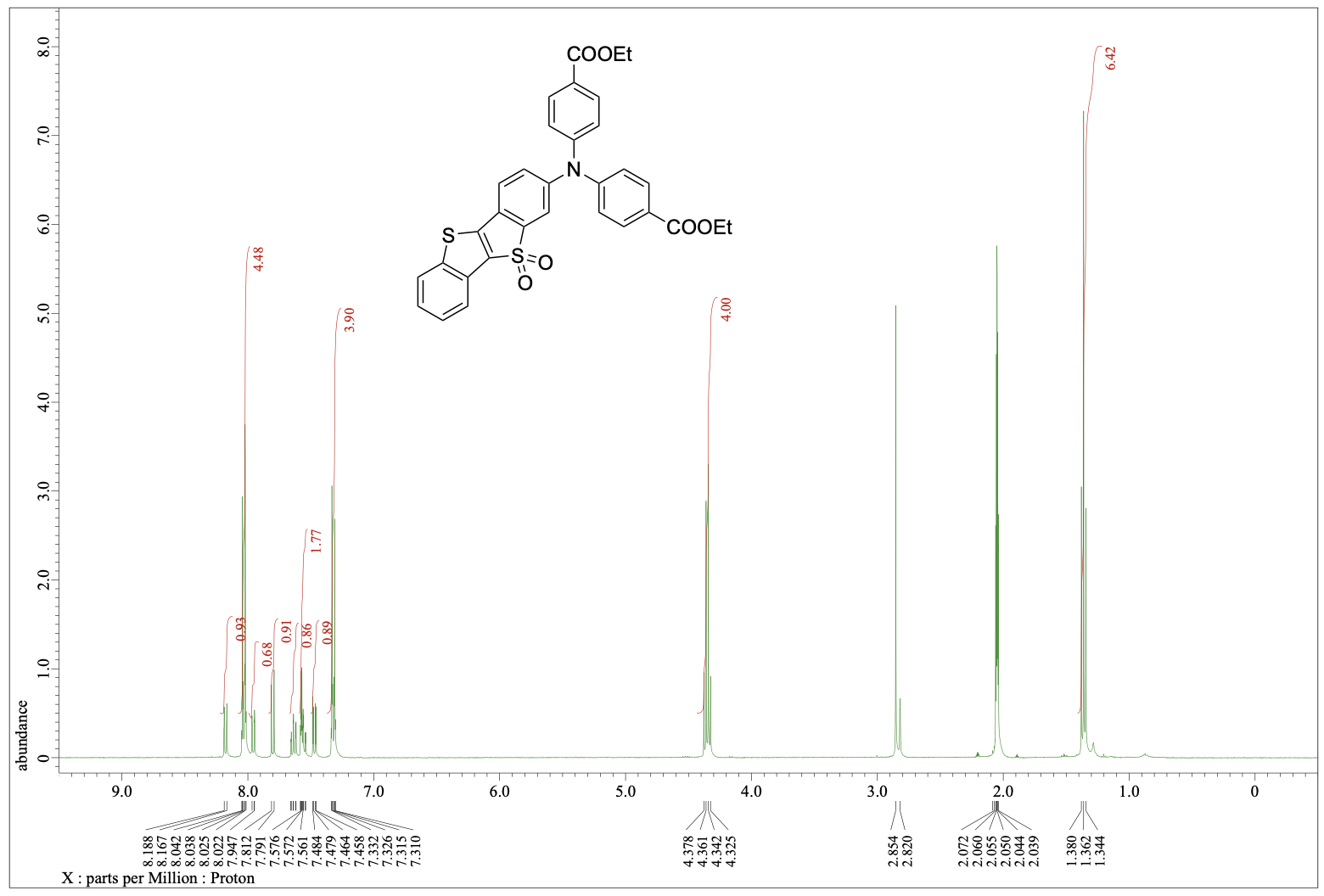


**Figure S16**. ^1^H NMR spectrum of **4** (400 MHz, acetone-*d*_6_).


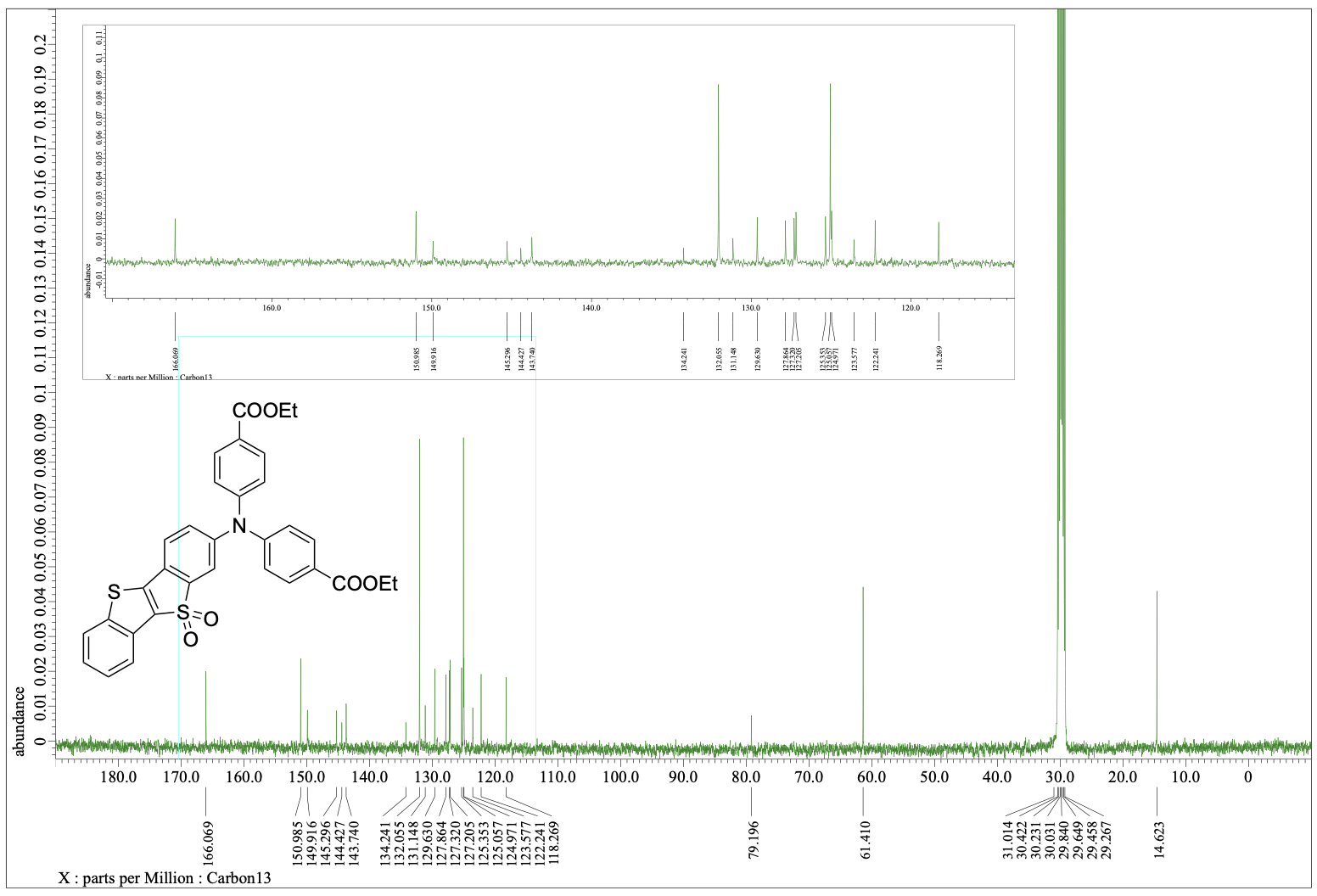


**Figure S17**. ^13^C NMR spectrum of **4** (100 MHz, acetone-*d*_6_).


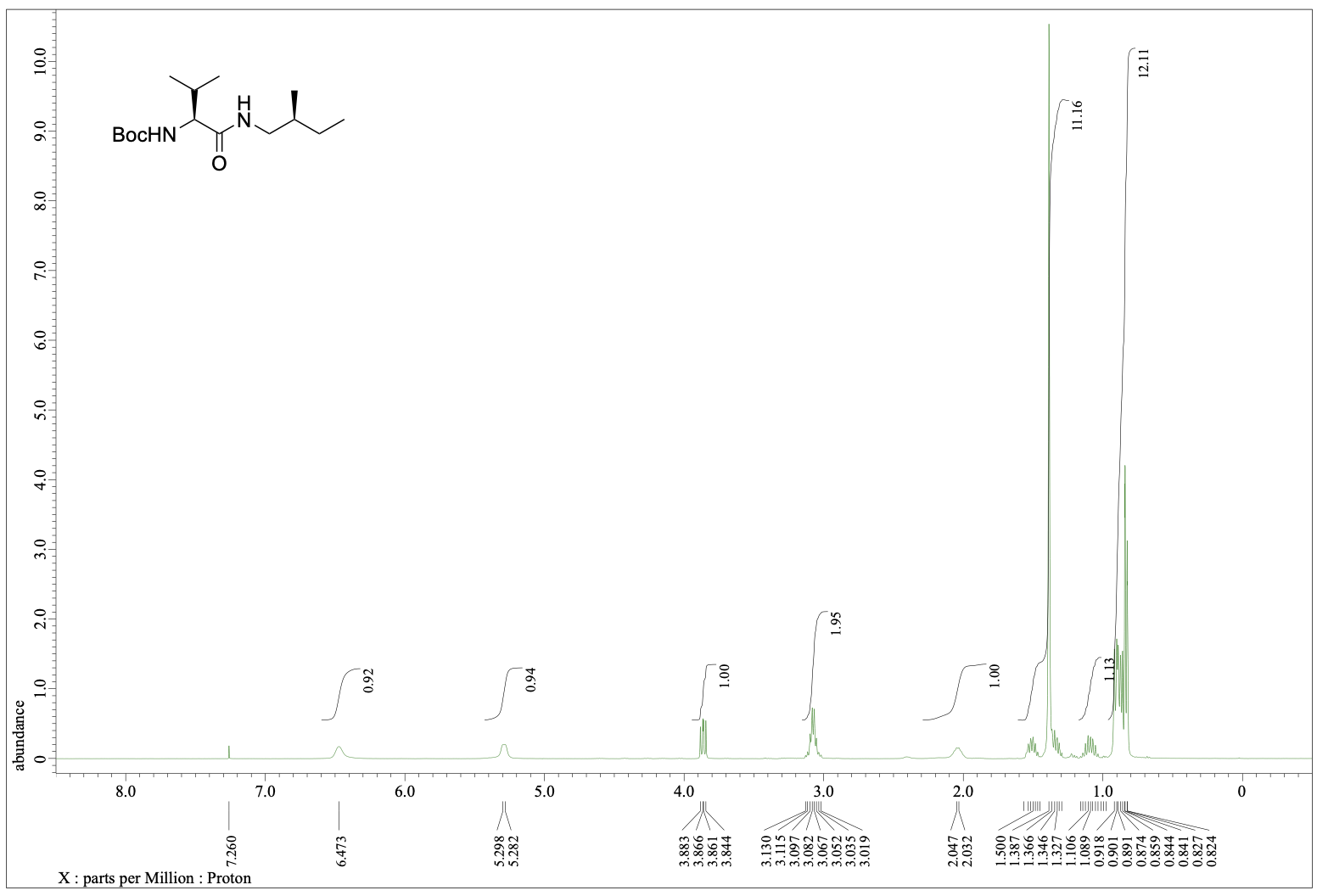


**Figure S18.** ^1^H NMR spectrum of **S2_Val_** (400 MHz, CDCl_3_).


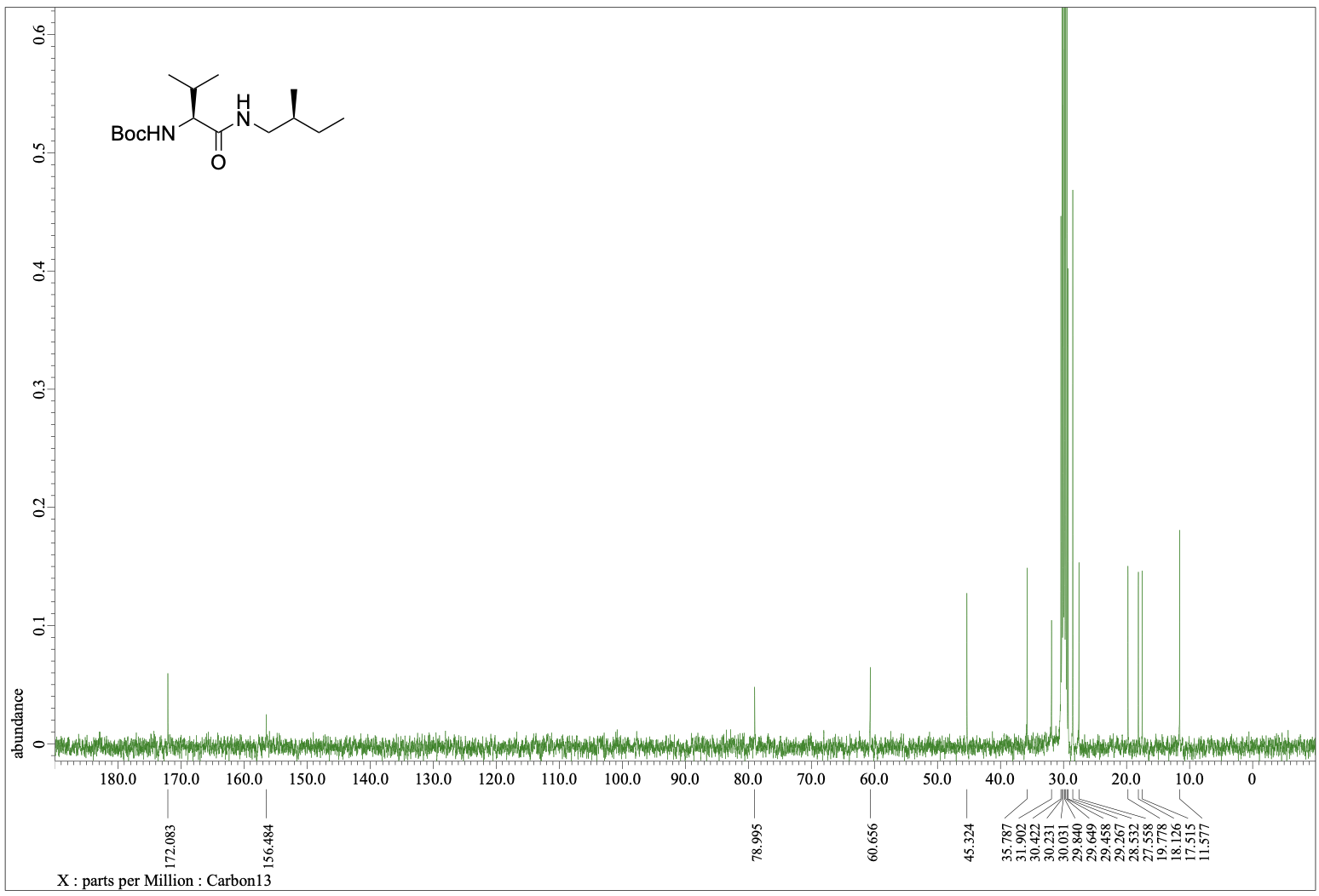


**Figure S19.** ^13^C NMR spectrum of **S2_Val_** (100 MHz, acetone-*d*_6_).


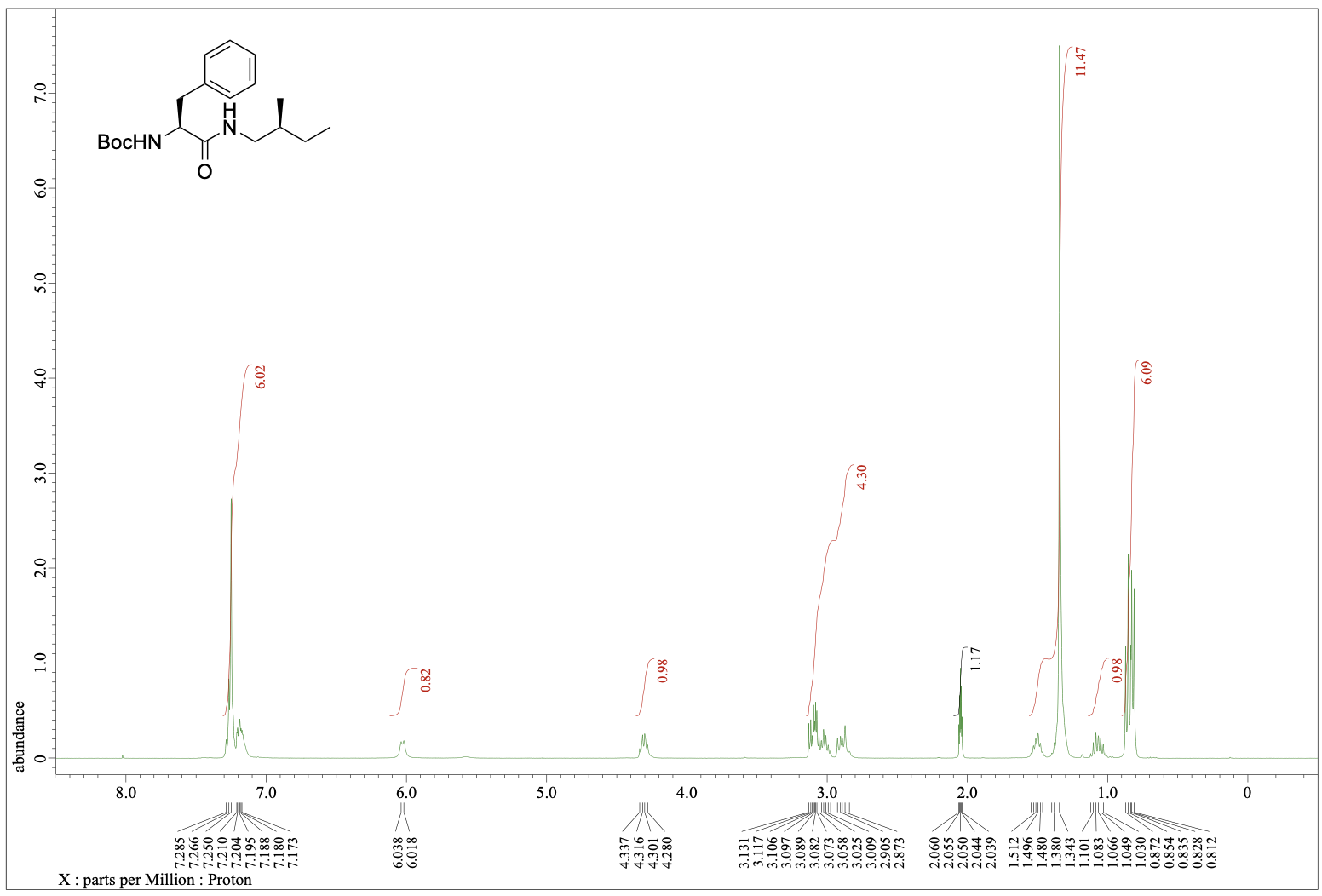


**Figure S20.** ^1^H NMR spectrum of **S2_Phe_** (400 MHz, acetone-*d*_6_).


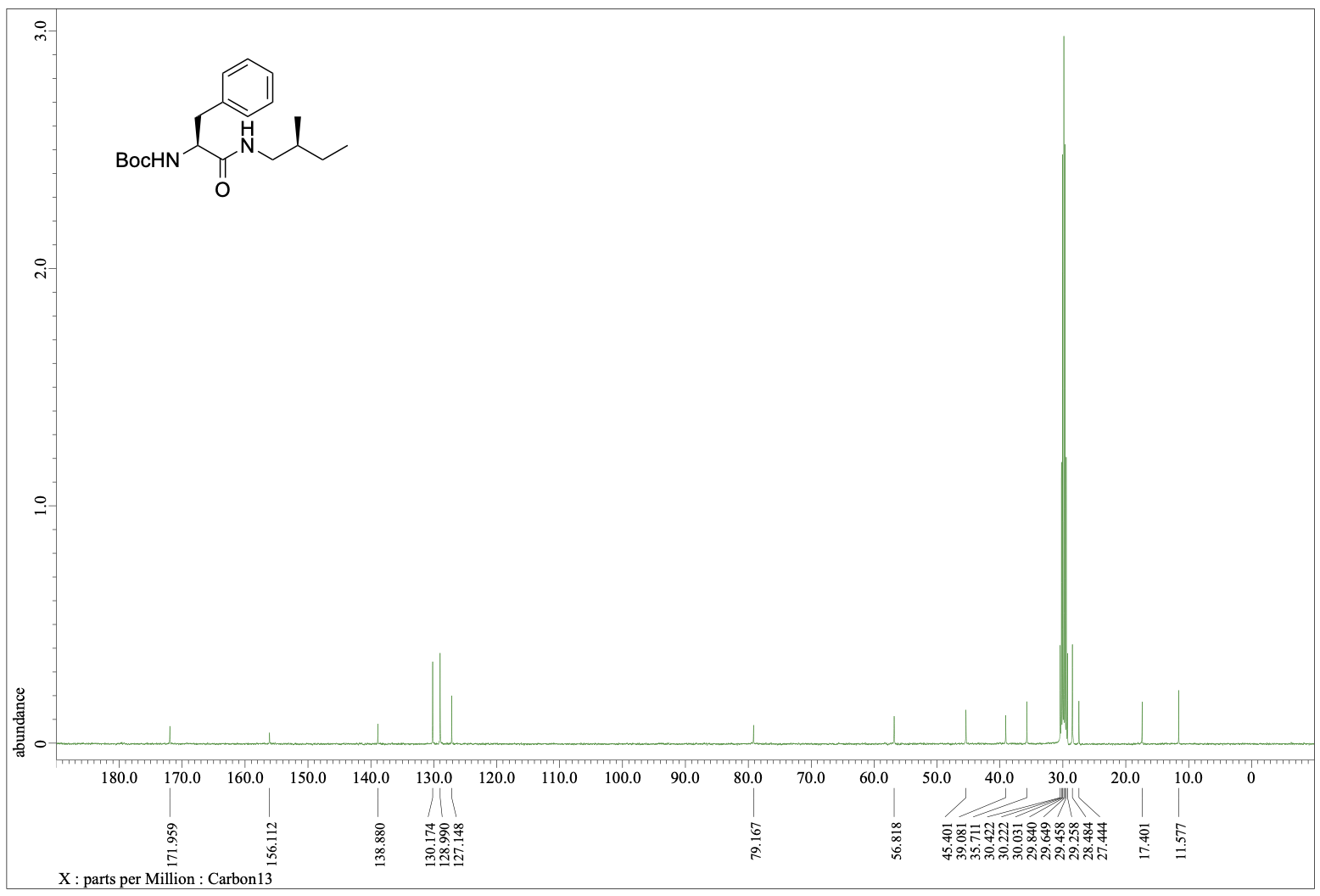


**Figure S21**. ^13^C NMR spectrum of **S2_Phe_** (100 MHz, acetone-*d*_6_).


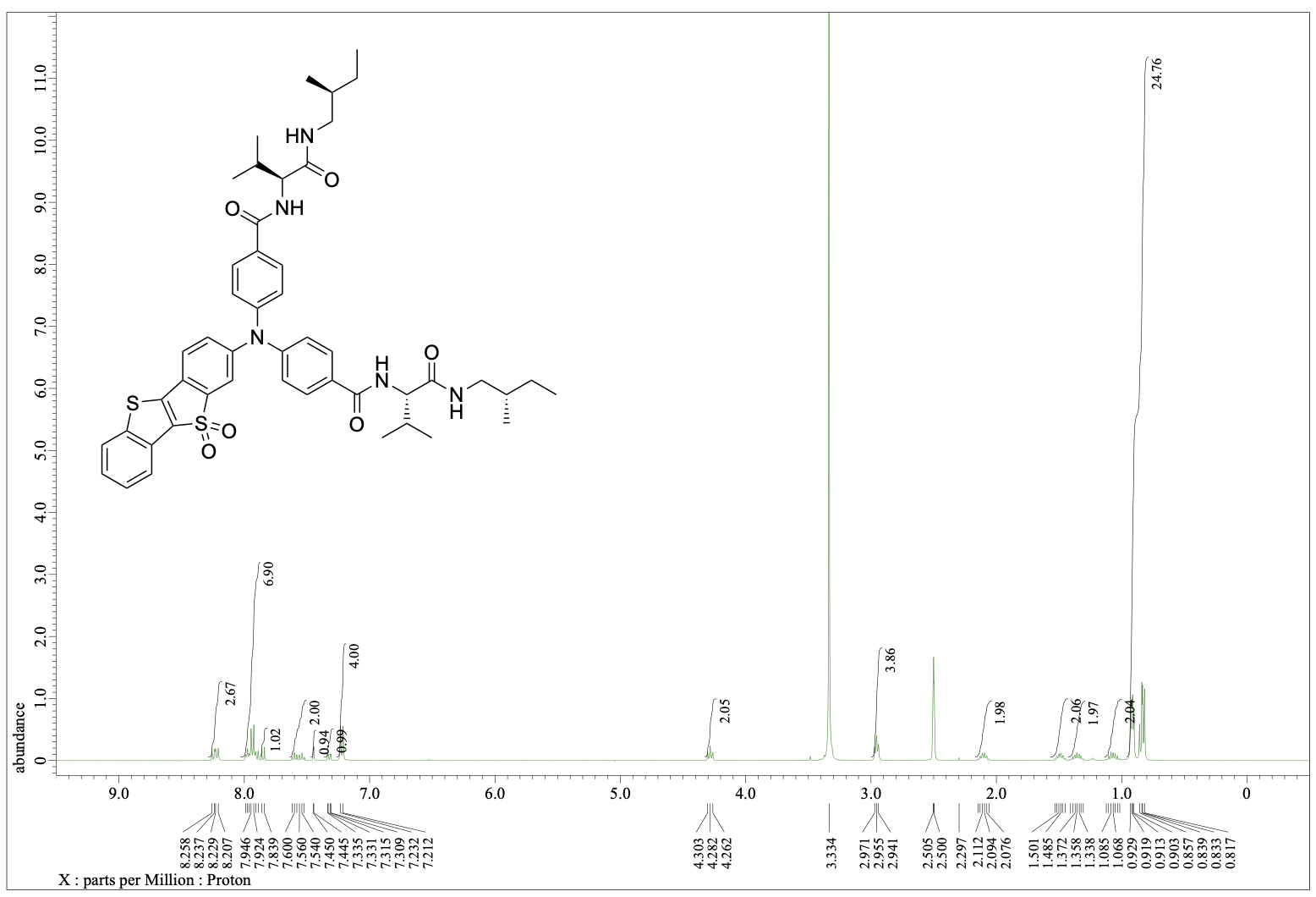


**Figure S22**. ^1^H NMR spectrum of **1_Val_** (400 MHz, DMSO-*d*_6_).


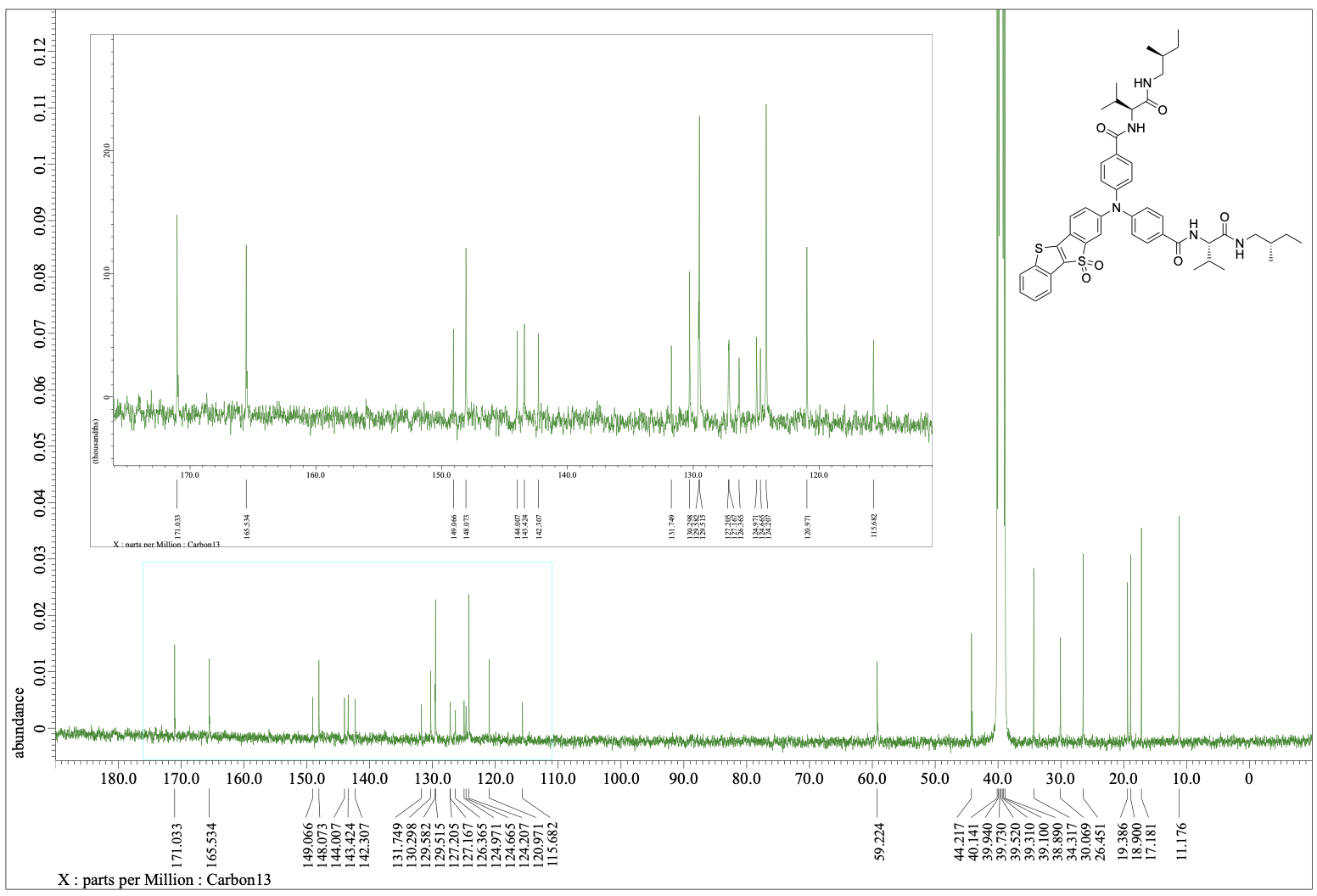


**Figure S23.** ^13^C NMR spectrum of **1_Val_** (100 MHz, DMSO-*d*_6_).


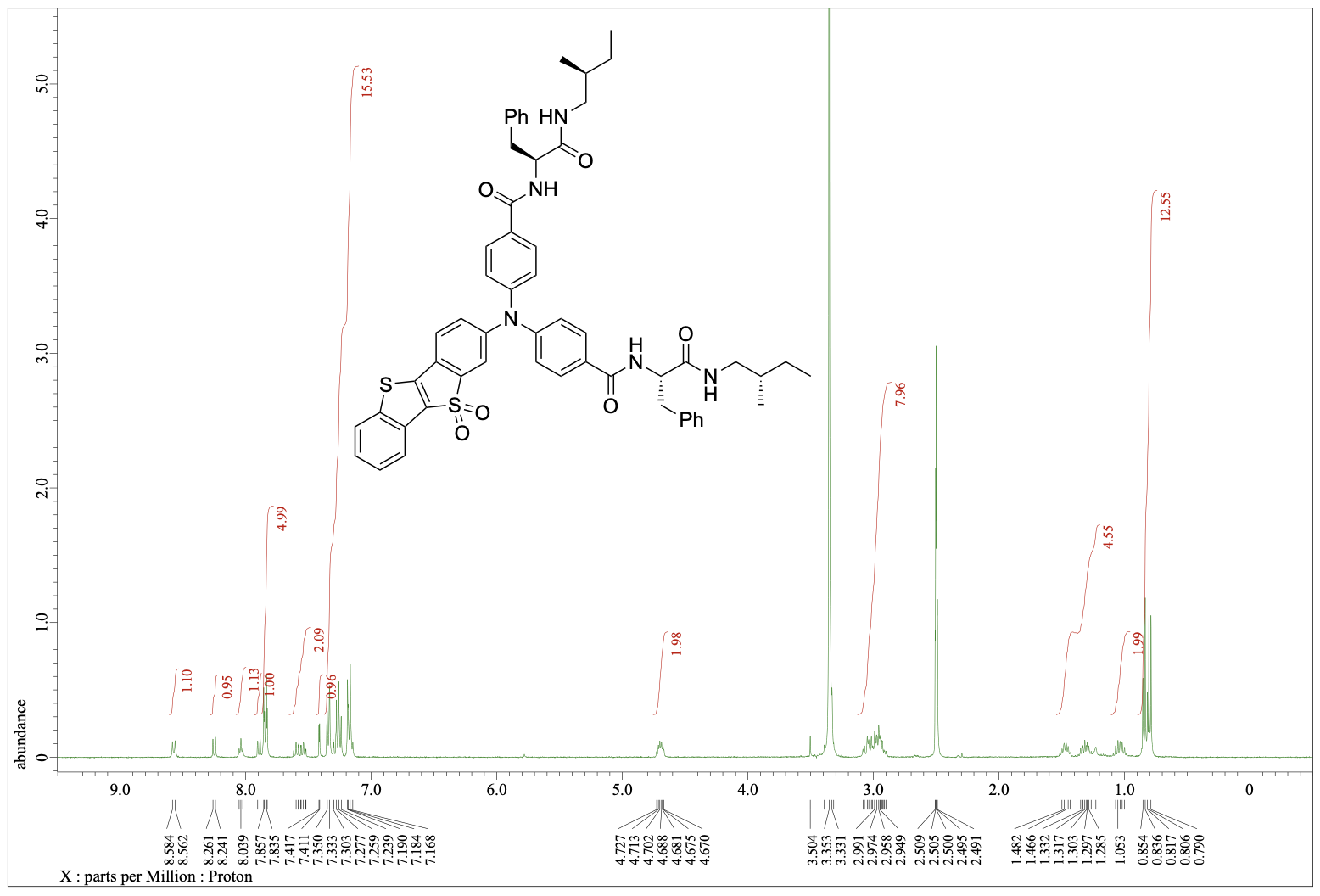


**Figure S24**. ^1^H NMR spectrum of **1_Phe_** (100 MHz, DMSO-*d*_6_).


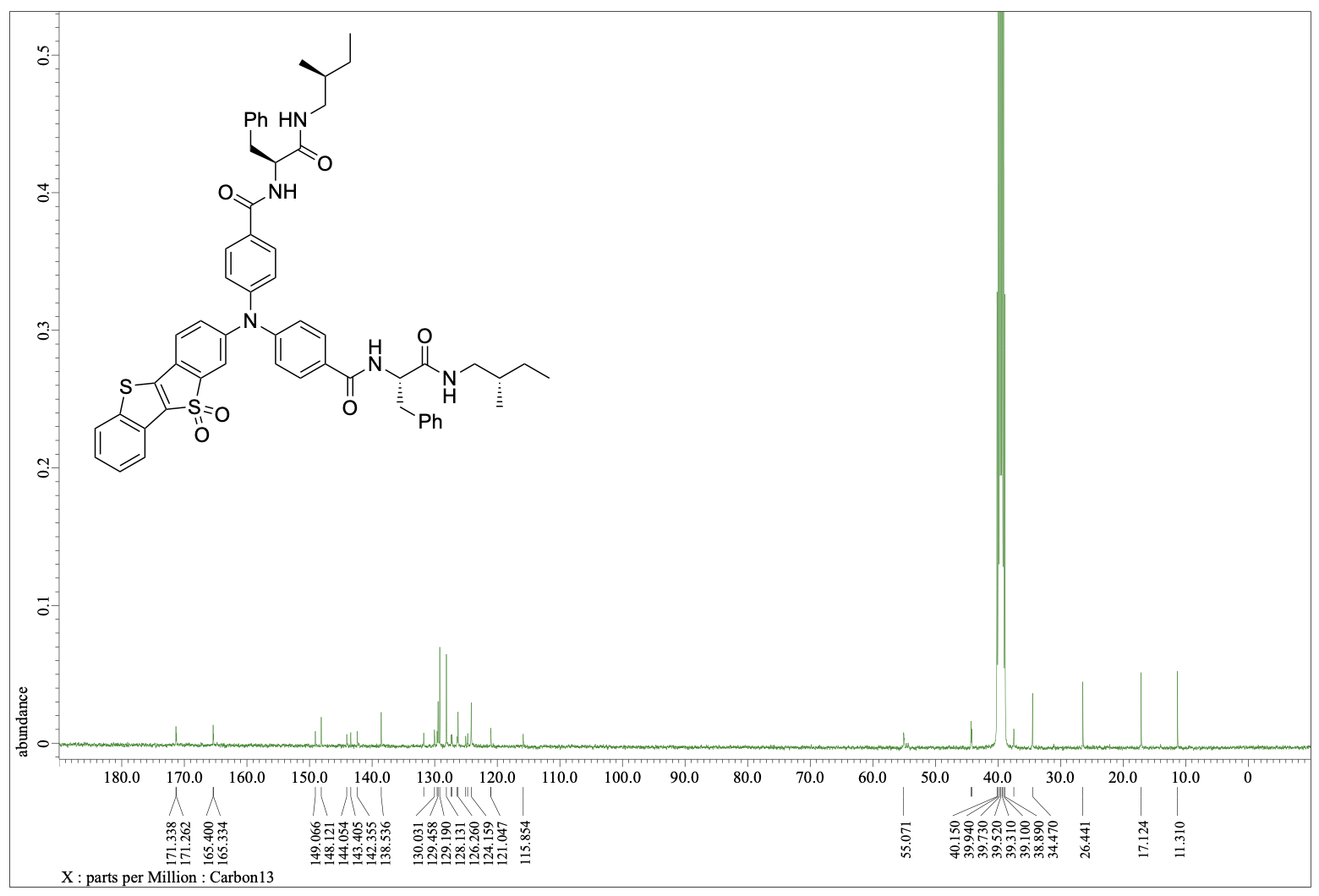


**Figure S25**. ^13^C NMR spectrum of **1_Phe_** (100 MHz, DMSO-*d*_6_).


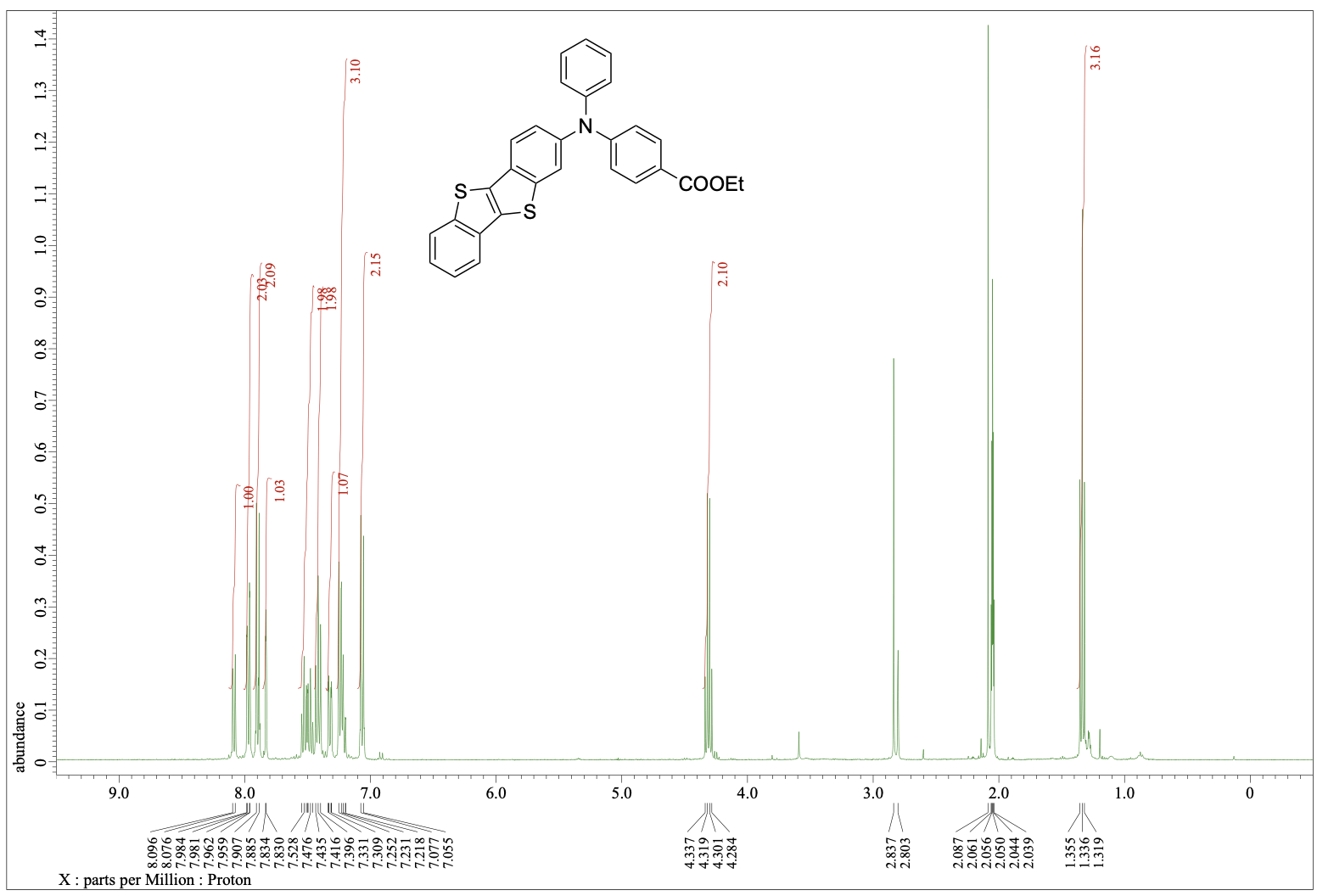


**Figure S26**. ^1^H NMR spectrum of **3*'*** (400 MHz, acetone-*d*_6_).


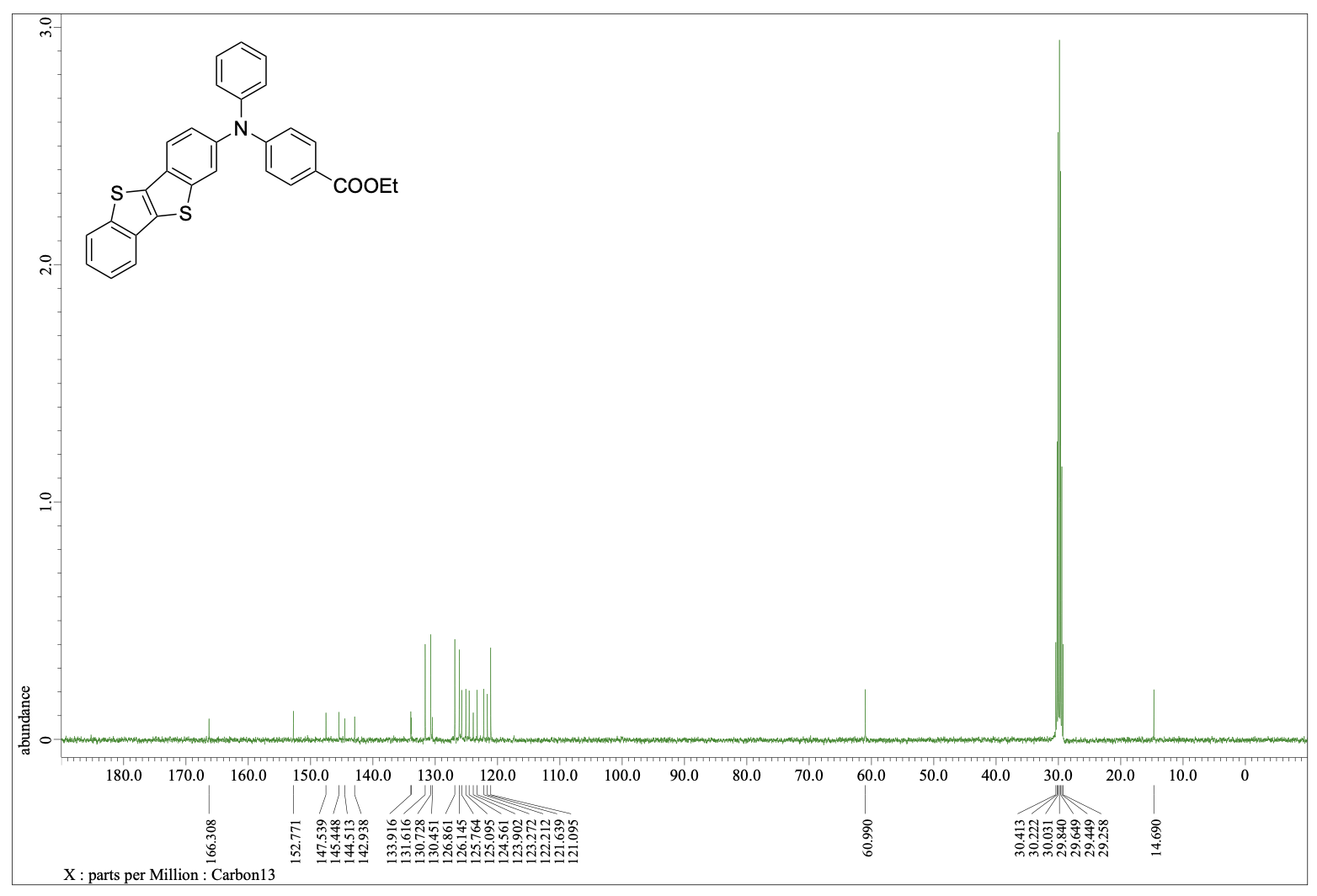


**Figure S27**. ^13^C NMR spectrum of **3*'*** (100 MHz, acetone-*d*_6_).


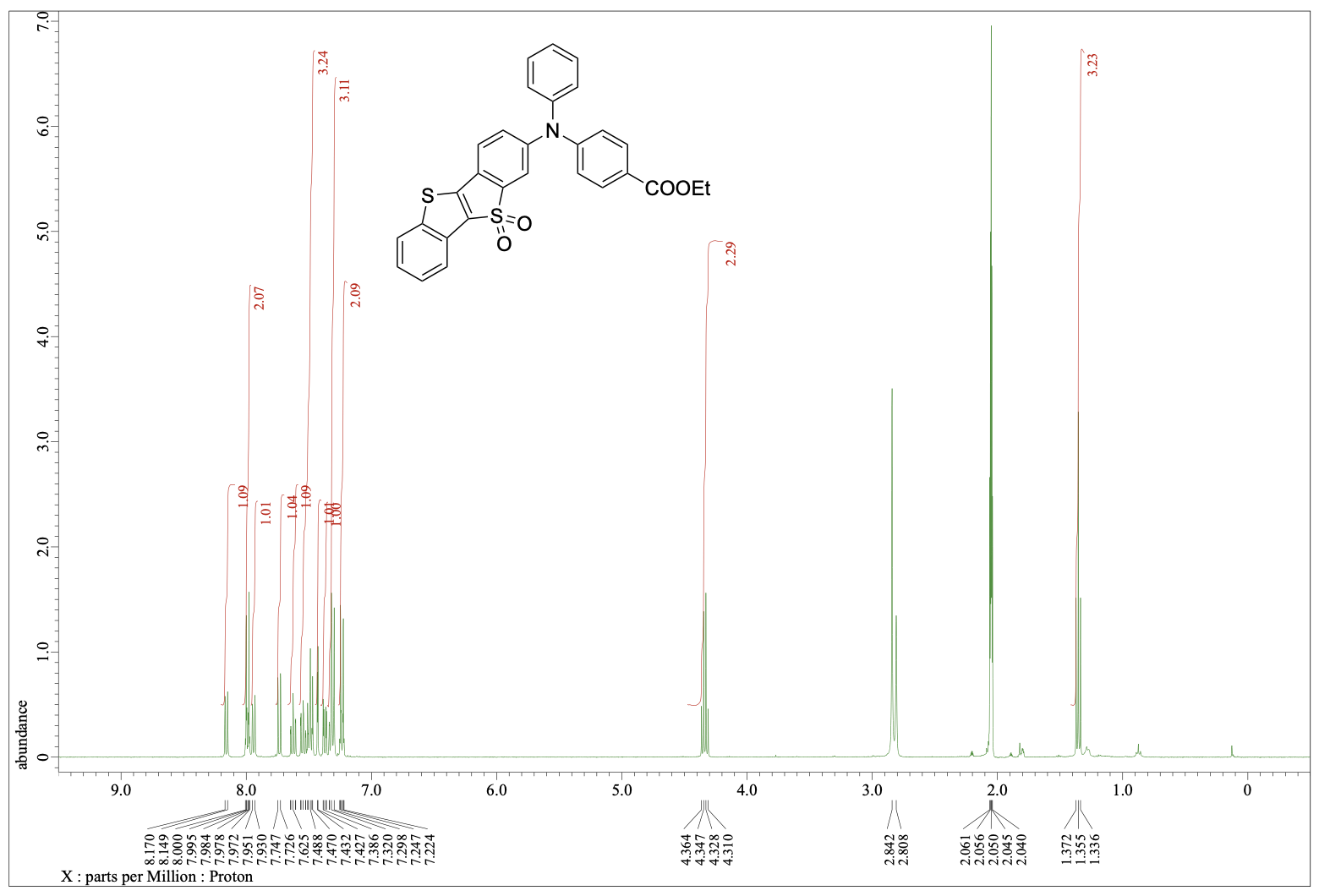


**Figure S28**. ^1^H NMR spectrum of **4*'*** (400 MHz, acetone-*d*_6_).


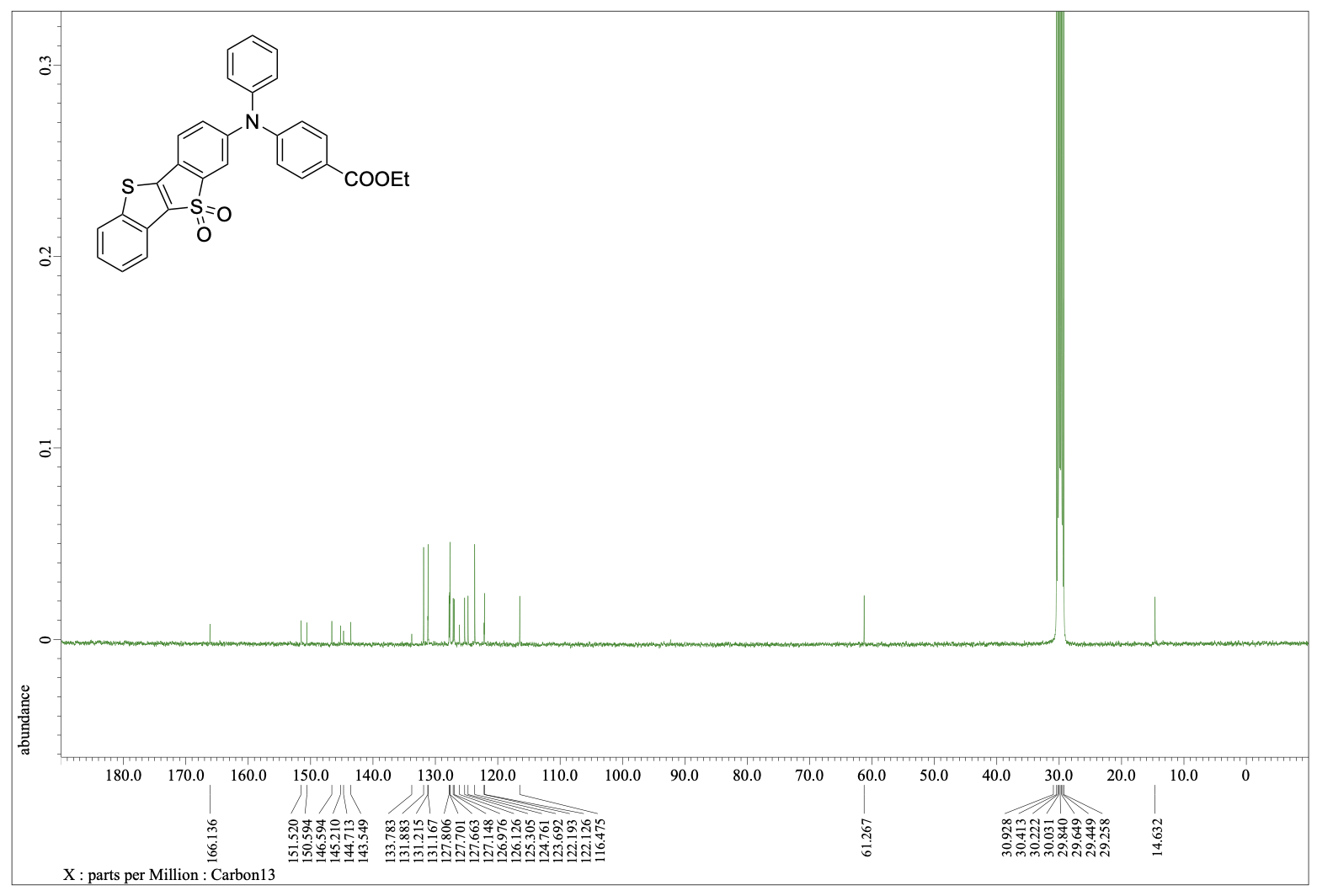


**Figure S29**. ^13^C NMR spectrum of **4*'*** (100 MHz, acetone-*d*_6_).


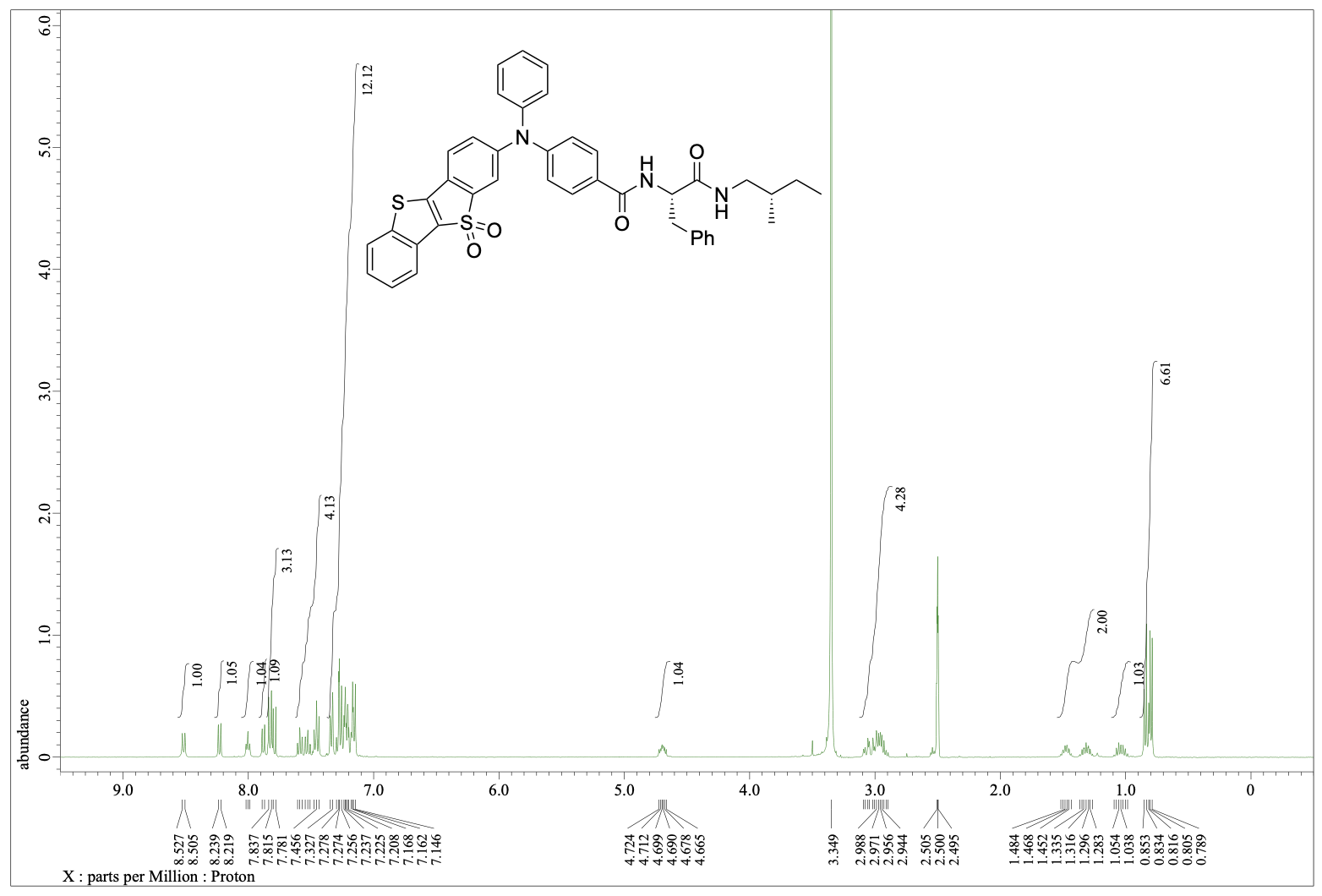


**Figure S30**. ^1^H NMR spectrum of **1_Phe_*'*** (400 MHz, DMSO-*d*_6_).


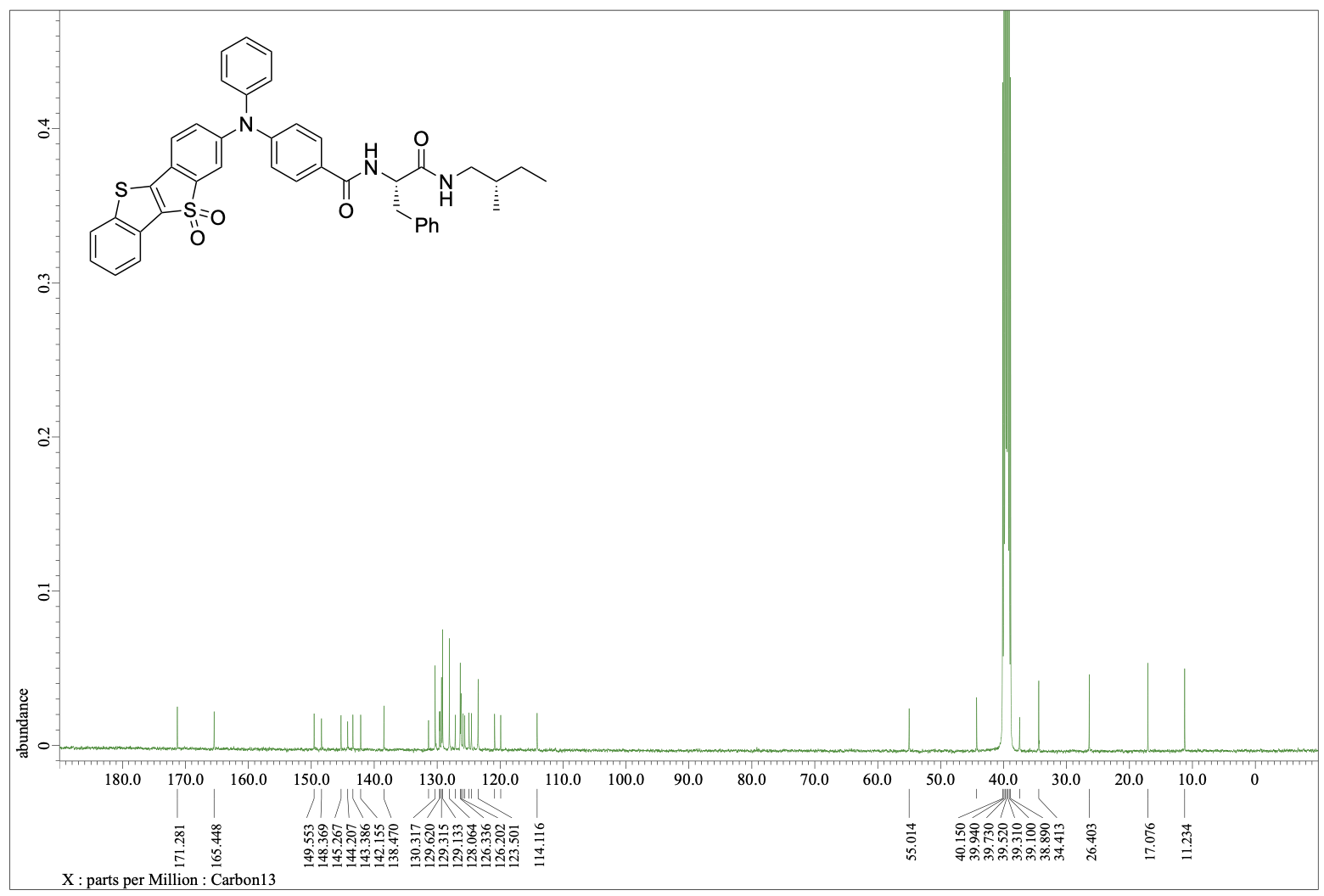


**Figure S31**. ^13^C NMR spectrum of **1_Phe_*'*** (100 MHz, DMSO-*d*_6_).
